# Determination of Multi-Residue of Brominated Phenols in Water, Sawdust Powder, and Fish Meal Samples by Preconcentration with Hydrophobic Menthol-Based Natural Deep Eutectic Solvent and HPLC-DAD

**DOI:** 10.1021/acsomega.6c03071

**Published:** 2026-08-04

**Authors:** Letícia Maria Effting, Thifany Miyuki Terossi Makita, João Victor Pastorin De Carvalho, Mariana Gava Segatelli, Cesar Ricardo Teixeira Tarley

**Affiliations:** Laboratório de Desenvolvimento de Métodos Analíticos (LADEMA), Departamento de Química, 37894 Universidade Estadual de Londrina (UEL), Londrina, Paraná 86057-970, Brazil

## Abstract

In this work, a menthol-based natural deep eutectic solvent (NADES) was prepared and applied for the liquid–liquid microextraction (LLME) of five brominated phenols (BPs) (2-bromophenol, 4-bromophenol, 2,6-dibromophenol, 2,4-dibromophenol, and 2,4,6-tribromophenol) from river and lake water, sawdust powder, and fish meal samples, with further determination by HPLC-DAD. NADES were characterized by using Fourier Transform Infrared Spectroscopy (FTIR), Differential Scanning Calorimetry (DSC), and Proton Nuclear Magnetic Resonance (^1^H NMR). Method optimization was performed using a 2^4^ factorial design and Doehlert design combined with the Derringer–Suich desirability function and response surface methodology, using the NADES produced with menthol and acetic acid. Optimal extraction conditions were achieved at a pH of 3.92, with 11.56% (w/v) NaCl, a vortex-assisted extraction time of 108 s, and 100 μL of NADES. Analytical performance parameters were established, yielding enrichment factors ranging from 90.2 to 120.8 and low limits of quantification (LOQs) between 0.49 and 1.47 μg L^–1^. Intraday (n= 5) and intermediate (n= 5) precision, expressed as relative standard deviation (%RSD), ranged from 1.14% to 5.11% at concentrations of 5.0, 25.0, and 75.0 μg L^–1^. The method was successfully applied to the analysis of BPs in river and lake water samples, as well as in sawdust powder and fish meal, using external calibration, and High-Performance Liquid Chromatography-Mass Spectrometry (HPLC-MS) was used to confirm the presence of 2-bromophenol, 4-bromophenol, and 2,6-dibromophenol in fish meal samples. The proposed method offers notable advantages, including high sensitivity, operational simplicity, and adherence to green chemistry principles due to the use of environmentally friendly solvents.

## Graphical abstract

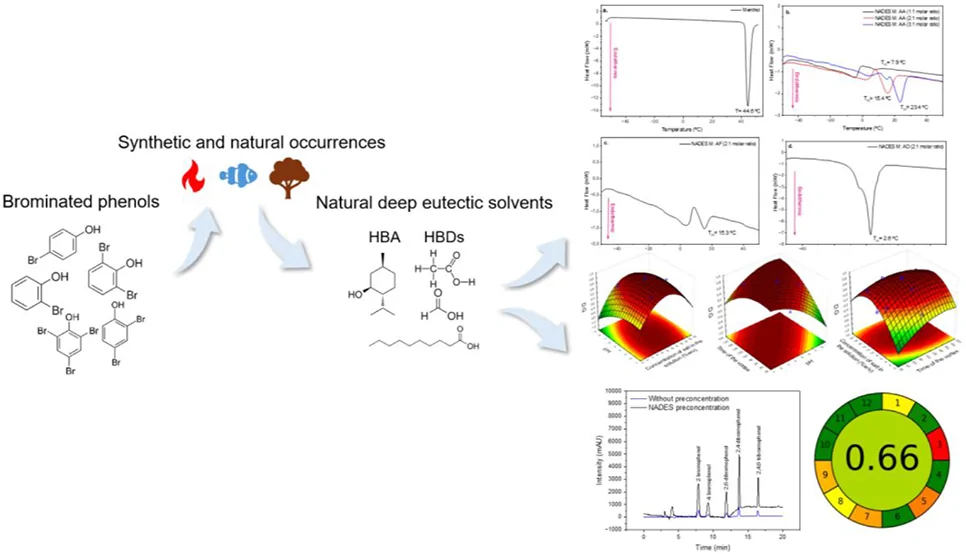

## Introduction

Brominated phenols (BPs) are characterized by hydroxyl groups and bromine atoms attached to a benzene ring, with their structural diversity resulting from variations in the number and position of bromine atoms. BPs have high logK_ow_, high lipophilicity, and relatively low-water solubility, being slightly acidic compounds.^1,2^ They can have either a natural or synthetic origin. 2-bromophenol (2-BP), 4-bromophenol (4-BP), 2,4-dibromophenol (2,4-DBP), 2,6-dibromophenol (2,6-DBP), and 2,4,6-tribromophenol (2,4,6-TBP) have been identified as key flavor compounds found in seafood.^3^ 2,4-DBP, 2,6-DBP, and 2,4,6-TBP are considered secondary metabolites in marine algae and have been detected in various aquatic organisms.^4,5^

4-BP was the first brominated phenol synthesized in the 1920s. Since then, several BPs have been synthesized, including 2-BP, 2,4-DBP, 2,6-DBP, and 2,4,6-TBP.^6^ These compounds have been used as flame retardants and in various applications, including pigments, herbicides, germicides, and antifungal agents. 2,4,6-TBP is used as a wood preservative due to its fungicidal properties. 2,4-DBP and 2,4,6-TBP are the primary intermediates for synthesizing tetrabromobisphenol A (TBBPA) and 1,2-bis­(2,4,6-tribromophenoxy)­ethane (BTBPE),^1,3,5^

Due to their widespread occurrence, both synthetic and natural BPs have been detected in various samples. This presence poses significant risks to both the environment and human health. BPs are suspected of acting as endocrine disrupters in humans.^2,5^ 2,4,6-TBP is a potentially carcinogenic, mutagenic, and toxic to reproduction and has persistent and bioaccumulative properties.^7^ BPs are not listed in any legislation, either regional or global, that establishes a maximum residue limit (MRL) for them. However, since 2,4,6-TBP can be classified as a wood preservative by the World Health Organization (WHO), in this case, the European Union applies a standard MRL for pesticides of 0.01 mg kg^–1^.^7^

Due to the potential adverse effects a variety of sample preparation methods, including solid-phase extraction (SPE),^1,8,9^ derivatization reaction with further liquid–liquid extraction (LLE),^10^ solid-phase microextraction (SPME),^11^ and simultaneous steam-distillation extraction (SDE),^12^ have been applied to the extraction/preconcentration of BPs in river and wastewater,^1^ seawater,^8^ water,^7,9^ urine,^10^ seafoods,^12^ and wood^13^ samples. The quantification of BPs has usually been carried out by high-performance liquid chromatography mass spectrometry (HPLC/MS),^1,9^ HPLC-DAD,^11^ or gas chromatography (GC) with electron capture detector (ECD)^8^ and mass spectrometry (MS) associated with a derivatization reaction.^10,12,13^

In recent decades, initiatives have been made to reduce or eliminate the use of toxic organic solvents in sample preparation. Green Analytical Chemistry (GAC) emerges as an area focused on designing and developing new analytical methods that reduce the use and generation of hazardous substances at every stage of chemical analysis,^14–16^ Although classical dispersive liquid–liquid microextraction (DLLME) partially meets the requirements of GAC. The use of chlorinated extraction solvents and organic disperser solvents remains the main bottleneck of the process.^17^

In this sense, microextraction methods employing environmentally friendly solvents, including supramolecular solvents (SUPRAs) and deep eutectic solvents (DES), have gained considerable attention.^18,19^

SUPRAs are formed through a sequential self-assembly process of amphiphiles, mainly long-chain alcohols or fatty acids, in the presence of a disperser agent (THF or acetonitrile), with water acting as a coacervation-inducing agent. DES are composed of low-toxicity mixtures of two or more compounds that interact through hydrogen bonding, with one component acting as a hydrogen bond acceptor (HBA) and the other as a hydrogen bond donor (HBD). These interactions significantly lower the melting point of the mixture compared with those of the individual constituents.^19–21^

Currently, DES derived from natural compounds provides particular interest, commonly referred to as natural DES (NADES). The advantages of these solvents stem from their favorable physicochemical properties, including low melting points, negligible volatility, nonflammability, low vapor pressure, tunable polarity, high chemical and thermal stability, and excellent miscibility and solvation capacity. Furthermore, their formation is based on intermolecular hydrogen-bond interactions rather than chemical reactions, eliminating the generation of byproducts and reducing the need for purification steps.^22^ As a result, their preparation is generally simple, efficient, and associated with minimal waste generation, making them ideal materials for developing sustainable analytical methods.^14^ For this reason, NADES have emerged as a more attractive class of sustainable solvents, aligning more closely with the concept of green chemistry.

The performance of NADES in extraction processes depends on the nature of the HBDs and HBAs, their molar ratios, and the intermolecular interactions established between them. These factors play an important role in the physicochemical properties, such as viscosity and polarity, thereby directly influencing extraction efficiency. NADES can be prepared from natural compounds, including salts, sugars, polyols, amino acids, terpenes, and organic acids.^23^ Recently, menthol has emerged as one of the most used HBAs due to its remarkable characteristics, such as biodegradability, nontoxicity, and low cost.^16^ Moreover, its molecular structure, characterized by a hydroxyl group and a hydrophobic cyclohexane moiety, confers an intrinsic capacity to efficiently extract nonpolar and moderately polar analytes.^14–16,24^

The first menthol-based NADES was described by Ribeiro and coworkers.^23^ Since then, menthol-based NADES have been successfully employed in the development of microextraction methods, offering low environmental impact due to their low toxicity, biodegradability, and high extraction selectivity.^14,16,25–27^

Several studies have reported the use of hydrophobic DES for the extraction of phenolic compounds, demonstrating satisfactory performance and considerable potential for microextraction procedures.^28–31^ However, some studies still rely on organic solvents in DES-assisted microextraction methods.^32,33^

According to the aforementioned and bearing in mind the absence of studies related to the preconcentration of BPs using green analytical approaches. This study focuses on the development of environmentally friendly menthol-based NADES using different HBDs for the preconcentration of 2-BP, 4-BP, 2,6-DBP, 2,4-DBP, and 2,4,6-TBP (Table S1). Menthol-based NADES were characterized by Fourier transform infrared spectroscopy (FTIR), differential scanning calorimetry (DSC), and proton nuclear magnetic resonance (H^1^ NMR). The optimization was carried out using a 2^4^ factorial design and Doehlert design combined with the Derringer–Suich desirability function and response surface methodology. Subsequently, the developed method was applied to analyze river and lake water, sawdust powder, and fish meal samples using external calibration.

## Materials and Methods

### Chemicals and Solutions

All reagents were of analytical or HPLC grade. Methanol (CH_3_OH, ≥99.9%), menthol (C_10_H_20_O, ≥99.9%), acetic acid (CH_3_COOH, ≥99.9%), formic acid (CH_2_O_2_, ≥99.9%), decanoic acid (C_10_H_20_O_2_, ≥99.9%), sodium chloride (NaCl, ≥99.5%), sodium sulfate (Na_2_SO_4_, ≥99%), boric acid (H_3_BO_3_, ≥99.5%), phosphoric acid (H_3_PO_4_, ≥99.9%) were purchased from Sigma-Aldrich (St. Louis, MO, USA). Brominated phenols (BPs) standards, 2-bromophenol – 2-BP (C_6_H_5_BrO, ≥99.5%), 4-bromophenol – 4-BP (C_6_H_5_BrO, ≥99.5%), 2,4-dibromophenol – 2,4-DBP (C_6_H_4_Br_2_O, ≥ 99.5%), 2,6-dibromophenol – 2,6-DBP (C_6_H_4_Br_2_O, ≥99.5%), and 2,4,6-tribromophenol – 2,4,6-TBP (C_6_H_3_Br_3_O, ≥99.5%), were all purchased from Sigma-Aldrich (St. Louis, MO, USA).

Stock solutions of BPs (50 mg L^–1^) were prepared in methanol for five BPs and stored in amber flasks in the freezer (−20 °C). The working solutions were prepared daily, diluted with ultrapure water (18.2 MΩ·cm) from an ELGA PURELAB purification system (Woodridge, IL, USA).

### Instruments

Solutions and pH samples were measured using a pH mobile digital pH meter (Metrohm 827, Herisau, Switzerland). The vortex stirrer (SCILOGEX MX-S, Rocky Hill, CT, USA) and centrifuge (QUIMIS 0222T2, Diadema, SP, Brazil) were utilized in the microextraction procedure for stirring and phase separation of the samples. A Quimis 164 ultrasonic bath (Diadema, SP, Brazil) (Frequency 50/60 Hz and capacity 2.4 L) was used for sample preparation.

NADES were analyzed by Fourier Transform Infrared (FTIR) spectroscopy on a KBr pellet (1% w/w). These analyses were performed on a Shimadzu spectrometer, from 4000 to 400 cm^–1^, with a resolution of 4 cm^–1^ and 64 scans. The liquid–solid phase transitions investigated by Differential Scanning Calorimetry (DSC) were determined in a temperature range of −50 °C/50 °C with a heating and cooling rate of 5 °C min^–1^. The samples were analyzed in 40 μL aluminum crucibles using a Shimadzu Model DSC-60 calorimeter.

^1^H Nuclear Magnetic Resonance (H^1^ NMR) spectra were recorded using a Bruker Ascend III spectrometer operating at 400 MHz for proton detection, equipped with 5 mm multinuclear probes. Deuterated chloroform (CDCl_3_, δH = 7.26 ppm) was used as the solvent, and tetramethylsilane (TMS, δH = 0.00 ppm) served as the internal standard. Spectral data were processed with MestreNova software, version 14.2.

### HPLC Analysis

Chromatographic analyses were performed using an HPLC system (Shimadzu, Kyoto, Japan) equipped with an LC-20AT pump, a CTO-20A column oven, and a DGU-20A degassing system. All chromatographic area values were calculated using LabSolutions software, LC Solution version 1.25 (Shimadzu, Tokyo, Japan). HPLC-MS analyses were performed using a liquid chromatograph coupled to a triple quadrupole model (LC-MS-8040, Shimadzu, Tokyo, Japan). Statistical analyses were performed using StatSoft Statistica 7.0 software (StatSoft, Tulsa, USA).

Chromatographic separation was performed using a C18 core-shell column (Phenomenex, 250 × 4.5 mm, particle size 5 μm) and a guard column (Phenomenex, 4.0 × 30 mm i.d., particle size 5 μm), oven temperature 30 °C, with a mobile phase consisting of a binary gradient of methanol and water (MeOH: H_2_O, v/v) at a flow rate of 0.6 mL min^–1^. The gradient consisted of MeOH: H_2_O (60:40, v/v) from 0.00 to 3.00 min, followed by MeOH:H_2_O (80:20, v/v). 3.00–15.00 min; MeOH: H_2_O (100:0, v/v), 15.00–22.00 min; MeOH: H_2_O (60:40, v/v), 22.00–27.00 min.^34^ The wavelengths were set at λ = 276 nm for 2-BP, λ = 281 nm for 4-BP, λ = 285 nm for 2,6-DBP, λ = 288 nm for 2,4-DBP, and λ = 297 nm for 2,4,6-TBP. The retention times of the BPs were 7.88 min for 2-BP, 9.22 min for 4-BP, 11.90 min for 2,6-DPB, 13.60 min for 2,4-DPB, and 16.35 min for 2,4,6-TBP using a diode array detector (DAD).

HPLC-MS analyses were performed using a liquid chromatograph coupled to a triple quadrupole model. A CLC-ODS C18 Kinetex Core–shell column (250 mm × 4.6 mm i.d., 5 mm in particle size) and a Phenomenex guard column (4.0 mm × 3.0 mm i.d., 5.0 mm in particle size) were used as the stationary phase. The mobile phase is a gradient composed of solvent A: 0.1% formic acid in methanol and solvent B: 0.1% formic acid in deionized water, at a flow rate of 0.6 mL min^–1^, with a column oven temperature of 30 °C. The gradient consisted of A:B (60:40, v/v), 0.00–3.00 min; A:B (80:20, v/v). 3.00–15.00 min; A:B (100:0, v/v), 15.00–22.00 min; A:B (60:40, v/v), 22.00–27.00 min. The sample injection volume was 10 μL. The MS/MS analysis was operated in positive ESI mode. Multireaction monitoring (MRM) was applied to the detection of the target compounds.

### NADES Preparation

The syntheses of the NADES were carried out using menthol as the HBA and acetic acid, formic acid, or decanoic acid as the HBDs. Menthol and HBDs were weighed at a suitable molar ratio, mixed in airtight reagent bottles, and stirred at 80 °C until a homogeneous and stable liquid was formed. Then, the NADES were cooled and stored at room temperature. First, menthol: acetic acid (M:AA), menthol: decanoic acid (M:DA), and menthol: formic acid (M:FA) were prepared at a molar ratio of 2:1 to evaluate the best HBD and HBA. This condition was selected based on our previous studies,^35^ in which the formation of a deep eutectic mixture was demonstrated. Subsequently, to determine the optimal molar ratio between the components (HBA:HBD), NADES were prepared at ratios of 1:1, 2:1, and 3:1.

### Microextraction Procedure Using NADES

Using the best composition of NADES formed by menthol (HBA) and HBD, in a centrifuge tube of 50.0 mL, the simultaneous extraction of BPs at a 50.0 μg L^–1^ concentration was carried out using 40.0 mL of standard solution at pH 4.00, buffered with 0.01 mol L^–1^ Britton-Robinson Buffer (BR Buffer) and containing 10% (m/v) of NaCl. The extraction was performed using 200 μL of NADES, followed by vigorous stirring for 60 s and subsequent centrifugation at 3000 rpm for 60 s. Afterward, approximately 20 μL of the supernatant (rich phase) was collected, diluted at a ratio of 2:1 (v/v) in methanol, and injected into an HPLC-DAD system by using a manual syringe injection. Figure 1 depicts the scheme of NADES-based microextraction.

**Figure 1:**
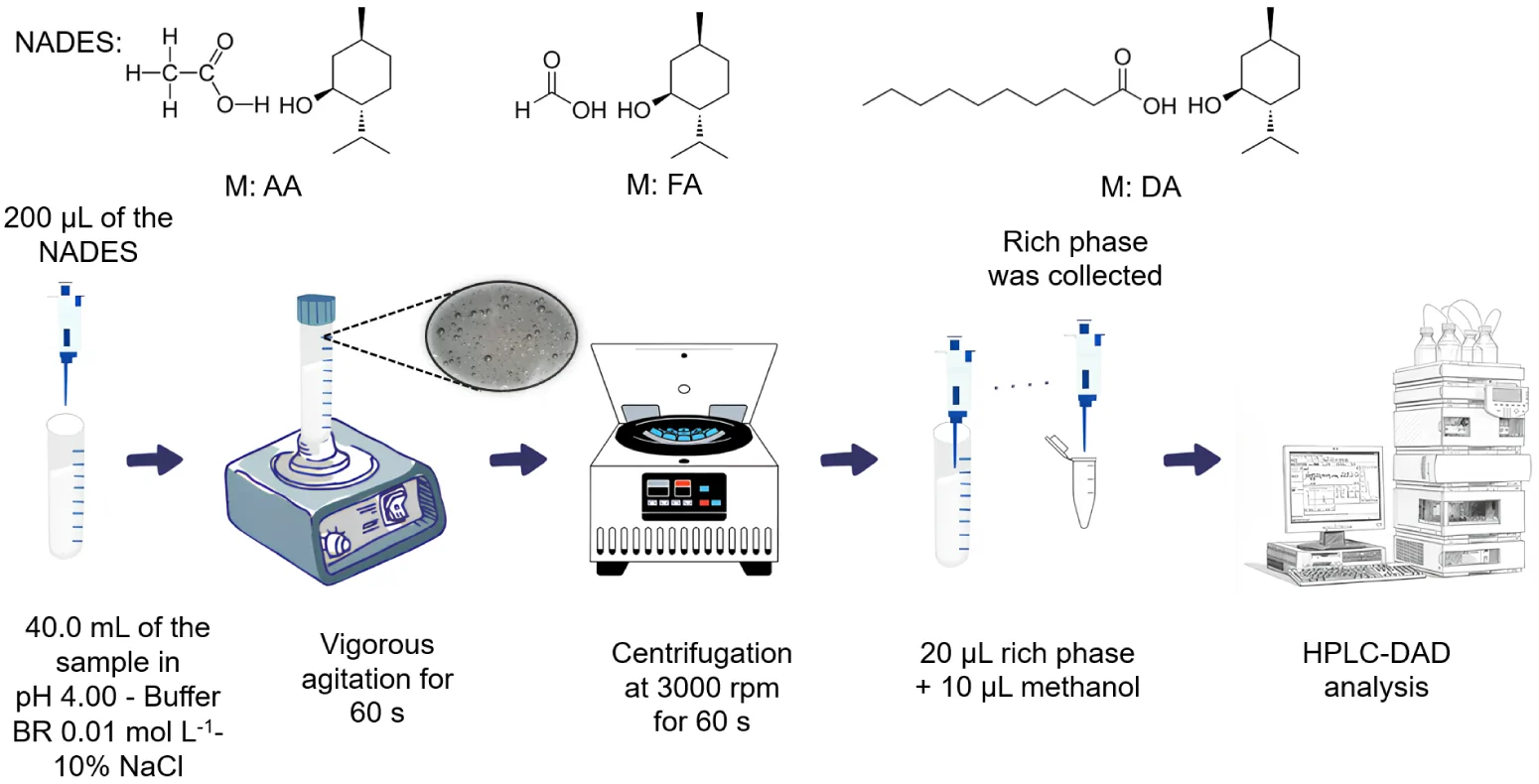
Schematic process of the liquid–liquid preconcentration method using NADES.

### Multivariate Optimization Procedure

The optimized condition of the proposed microextraction method was established using a 2^4^ factorial design and a Doehlert design,^35^ combined with the Derringer–Suich desirability function for multiresponse optimization^36^ and response surface methodology.

For the 2^4^ factorial design, the following factors and their levels were investigated: sample pH (4.00 and 8.00 buffered with BR Buffer at 0.01 mol L^–1^), vortex time (30 and 60 s), volume of the NADES (100 and 200 μL), and concentration of NaCl in the solution (0% and 10% w/v). The preconcentration volume was set at 40.0 mL, and the concentration for the BPs was 50.0 μg L^–1^. All experiments were performed in duplicate.

Doehlert design for those the most important factors was performed as follow: concentration of NaCl in the solution (2%, 6.5%, 11%, 15.5%, and 20% w/v), sample pH (2.00, 3.00, 4.00, 5.00, 6.00, 7.00, and 8.00 buffered with BR Buffer at 0.01 mol L^–1^), and vortex time (30, 105, and 180 s). The experiments were performed in triplicate at the central point.

The global desirability, adopted as the multiresponse for the experimental design (factorial and Doehlert design), was calculated based on the determination of the individual desirability functions (d^i^). The scale of the individual desirability function (*di*) ranges from *d* = 0, for a completely undesirable response, to *d* = 1, for a completely desirable response. To maximize the response (target value as the most desirable response), the one-way transformation according to eq 1 was applied:

1 $$\begin{array}{r} \mathrm{di}=0,\;{\mathrm{if Y}}_{i}\leq L\\ d_{i}={\left(\frac{Y_{i}-L}{H-L}\right)}^{r}\\ {\mathrm{di}=1,\;\mathrm{if}\;Y}_{i-}\geq L \end{array}$$

where *Y i* is the test response, *r* is the weight, *L* and *H* are the most undesirable and desirable responses of all tests, respectively. From the individual desirability, the global desirability was determined as the geometric mean according to eq 2.

2 $$Dg=\sqrt[{n}]{d_{1}^{p1}d_{2}^{p2}d_{3}^{p3}\cdot \cdot \cdot d_{m}^{p}}$$

where *m* is the number of responses studied for each experiment during the optimization process, and *n* is the weight value, which was 1 for all BPs. The influence of the factors on the global desirability was evaluated using a Pareto chart at a confidence interval of 95.0%.

### Analytical Parameters of NADES-Based Microextraction

The analytical parameters, including external linear calibration curve, limits of detection (LOD) and quantification (LOQ), enrichment factor (EF), as well as intraday and intermediate precision, were determined under optimized conditions. The LOD and LOQ were determined in accordance with IUPAC,^37^ using *3SD/m* and *10SD/m*, respectively, where SD is the standard deviation from ten blank measurements, and m is the slope of the calibration curve obtained after preconcentration with NADES.

One should note that LOD and LOQ are theoretical values, therefore, to ensure the practical feasibility of the values, standard solutions of the BPs at the LOQ concentration were prepared, subjected to the preconcentration step, and included as the first concentration point of the analytical curve. The validation of the linear equation obtained from the calibration curve was evaluated from analysis of variance (ANOVA) at a 95.0% confidence level.

The EF was determined by comparing the slopes of calibration curves with and without the preconcentration step. Precision was determined in terms of intraday repeatability and intermediate precision over two consecutive working days, using standard BPs solutions at concentrations of 5.0, 20.0, and 75.0 μg L^–1^ (n= 5) and assessed as relative standard deviations (RSD, %).^38^

### Determination of BPs in River and Lake Water, Sawdust Powder, and Fish Meal Samples

Water samples from lakes and rivers were collected from two different streams located in cities of Londrina (Igapó Lake, coordinates: 23°19′15.2 ″S 51°10′54.9 ″W), and Arapongas (Bandeira do Norte River, coordinates: 23°39′06.8 ″S 51°43′42.9 ″W) in Paraná State, Brazil. These cities are in regions known for their timber industry.

All samples were collected in dark glass containers, and their pH was adjusted to 2.00 using concentrated sulfuric acid. They were then filtered through 0.45 μm nitrate cellulose membranes (GVS Filter Technology, Morecambe, UK) to eliminate suspended solids and stored in a refrigerator, protected from light, until analysis. Before microextraction, both pH and salt concentration were adjusted. To assess the accuracy of method, samples were fortified with known concentrations (5.0, 20.0, and 75.0 μg L^–1^) of BPs and processed according to the optimized proposed procedure. These experiments were carried out in triplicate.

The sawdust powder was collected from a custom furniture company located in Londrina, Paraná, Brazil. The fish meal was purchased from a local supermarket in Londrina, Paraná, Brazil. The extraction of BPs from the samples (sawdust powder and fish meal) was ultrasound-assisted.^39,40^ For this task, 100 mg of sample was mixed with 50.0 mL of a 0.01 mol L^–1^ BR buffer solution at pH 3.92 and containing 11.56% NaCl for 20 min. The extraction was sequentially repeated five times. The extraction medium employed is identical to that used in the NADES-based microextraction step, as will be discussed later. Therefore, once the BPs have been leached from the samples, the extract can be directly subjected to microextraction without the need for pH adjustment.

In triplicate, 40.0 mL of extracted sawdust powder and fish meal was submitted to the microextraction procedure. The accuracy of the method was evaluated through spiking and recovery tests, in which samples were fortified with three concentrations (10.0, 40.0, and 150.0 mg kg^–1^) of the BPs.

## Results and Discussion

### NADES Characterization

FTIR and DSC were used to characterize the precursors and NADES, while H^1^ NMR was also employed to investigate the stability of NADES upon microextraction by the analysis of the rich phase.

Figure 1Sa shows the FTIR spectra for menthol, acetic acid, NADES M:AA (1:1 molar ratio), NADES M:AA (2:1 molar ratio), and NADES M:AA (3:1 molar ratio). Figure 1Sb shows the FTIR spectra for menthol, formic acid, and NADES M:FA (2:1 molar ratio), and Figure 1Sc shows the spectra for menthol, decanoic acid, and NADES M:DA (2:1 molar ratio).

The FTIR spectra of the HBD and menthol show a broad peak at approximately 3250 cm^–1^, which corresponds to its hydroxyl group (−OH), and the peaks in the range of 2950 to 2870 cm^–1^ correspond to a C–H sp^3^ bond. On the other hand, the FTIR spectra of the HBDs (acetic acid, formic acid, and decanoic acid) show the band close to 1730 cm^–1^ attributed to the carboxylic acid group.^24,41,42^

FTIR spectra of the eutectic mixtures indicate the intermolecular hydrogen bond interaction between the HBD and menthol, mostly in the hydroxyl group (−OH) region. The spectrum of NADES shows that the peak at 3250 cm^–1^ from menthol becomes broadened due to H-bond formation with the HBD molecules. In addition, the carboxylic group band of the HBD in the NADES was shifted to a lower wavenumber (∼1710 cm^–1^), further indicating the formation of new hydrogen bonds between the HBD and menthol.^24,41,42^

DSC analyses were performed to determine the melting points (Tm) of the pure menthol and the NADES. Figure 2 presents the DSC thermograms for menthol, NADES M:AA (1:1, 2:1, and 3:1 molar ratios), NADES M:FA (2:1 molar ratio), and NADES M:DA (2:1 molar ratio). As can be observed, the melting temperature (Tm) of the NADES shifted toward lower values compared to pure menthol, confirming the formation of eutectic mixtures. These outcomes corroborate the FTIR data, confirming the strong interactions between the HBA (menthol) and HBD (acetic acid, formic acid, and decanoic acid).^24,43^ Moreover, for the NADES formed by M:AA, it was observed that the higher the menthol content, the higher the melting temperature (Tm). The lower Tm (2.6 °C) was achieved for the NADES M:DA, and such a result can be attributed to the similar polarity of menthol and dodecanoic acid, which promotes stronger affinity and greater stability, as will be further demonstrated by the ^1^H NMR spectra.

**Figure 2:**
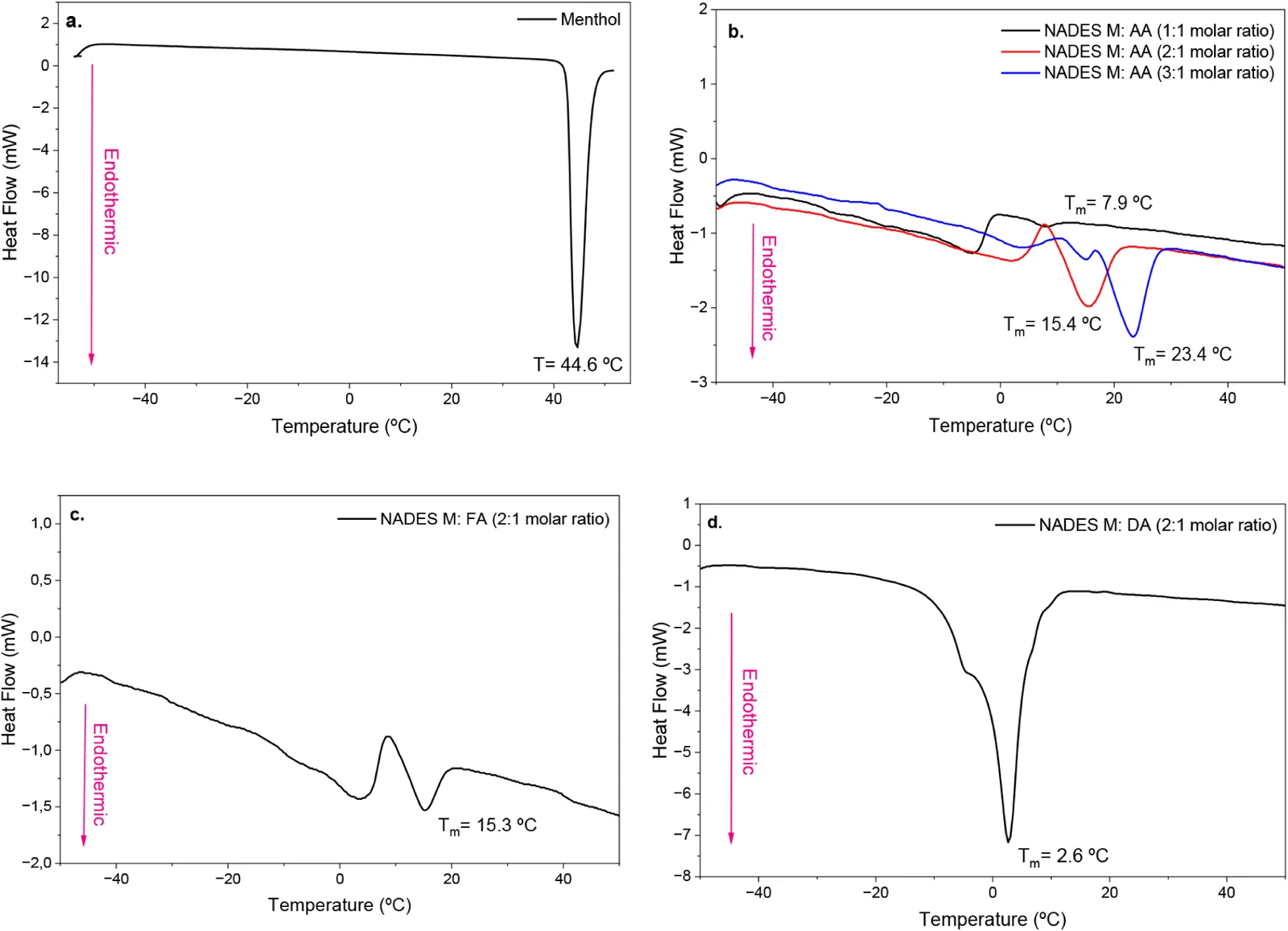
DSC thermogram of a. menthol, b. NADES M:AA (1:1, 2:1, and 3:1 molar ratio), c. NADES M:FA (2:1 molar ratio), and d. NADES M:DA (2:1 molar ratio).

^1^H NMR spectra of NADES M:AA (2:1 molar ratio), M:FA (2:1 molar ratio), M:DA (2:1 molar ratio), its pure components, and the rich phase are shown in Figure 2Sa, b, and c. The characteristic peaks of menthol (δ = 0.80–3.40 ppm), acetic acid (δ = 2.09 ppm), formic acid (δ = 8.25 ppm), and decanoic acid (δ = 0.85–2.86 ppm) were observed. The peaks of the NADES exhibit the characteristic signals of their pure reagents.^44^

On the other hand, in the ^1^H NMR spectrum of the rich phase upon the extraction step with the NADES M:AA (1:1, 2:1, and 3:1 molar ratio) (Figure 2Sa) the absence of the acetic acid peaks (δ = 2.09 ppm) was observed, indicating that only menthol acts as the extracting solvent, while the acids remain in the aqueous phase. However, the ^1^H NMR spectrum of the rich phase of the NADES M:FA (Figure 2Sb), and M:DA (Figure 2Sc) showed peaks corresponding to the acid (formic acid, δ = 8.25 ppm, and decanoic acid, δ = 0.85–2.86 ppm), thereby demonstrating the greater stability of these NADES, even under vigorous stirring in an aqueous medium.^44^

Some studies in the literature have already reported the disruption of hydrogen bonds between the constituents of NADES. Florindo and co-workers^44^ point out that stable NADES in water are achieved only when both components exhibit hydrophobic characteristics.

In the case of the M:AA NADES, it is estimated that approximately 90% of the acetic acid remains in the aqueous phase, resulting in a phase rich in menthol. In other NADES systems (i.e., M:FA), the transference of the acid into the aqueous phase is lower, and in some cases, when both components are hydrophobic (i.e., M:AD), no acid transference toward the aqueous medium from the NADES is observed.^45^

Based on the obtained results and considering the formation of a menthol-rich phase in the M:AA NADES, it can be suggested that menthol alone may extract the analytes in amounts similar to those achieved with the original NADES. However, as previously reported in the literature^15^ and supported by our results, menthol becomes liquid in water only at its melting point (44.6 °C; Figure 2a), representing a clear disadvantage compared to the use of NADES. NADES, in turn, require only the initial mixing of the two components for their formation and can be stored and used in various extraction procedures. Moreover, studies indicate that the presence of acetic acid (hydrophilic) facilitates the dispersion of menthol (hydrophobic) in water, thereby enhancing analyte extraction into the menthol-rich phase after acetic acid leaching.^14^

### Composition of the NADES

Three hydrophobic NADES were investigated for the preconcentration of BPs. Figure 3S shows the best composition of the NADES for the microextraction procedure. The components of the NADES greatly influence preconcentration efficiency. The NADES formed by M:AA (2:1 molar ratio) and M:FA (2:1 molar ratio) showed, in general, slightly higher extraction of BPs, compared to the NADES M:DA (2:1 molar ratio), which indicates that increasing the chain length of the HBD (decanoic acid) enhances the hydrophobicity of the solvent, making the extraction of the compounds more difficult. Additionally, the higher viscosity hinders the mass transfer of the analytes to the extraction phase.^39^ As the NADES M:AA showed slightly higher extraction efficiency for all target analytes, it was selected for further experiments.

The superior extraction efficiency observed for the NADES M:AA (2:1 molar ratio) may be attributed to its intermediate polarity compared with the NADES M:DA (2:1 molar ratio). Considering the intermediate Log K_ow_ values of the investigated BPs (Table S1), these compounds exhibit a greater tendency to partition from the aqueous phase into the NADES phase, thereby enhancing extraction efficiency. Furthermore, hydrogen-bonding interactions may contribute to analyte stabilization within the NADES phase. The phenolic hydroxyl groups of the BPs can interact with both the hydroxyl group of menthol and the carboxylic group of acetic acid, which act as HBD and HBA sites within the eutectic network.^46^

The molar ratio between menthol and acetic acid was also evaluated, as shown in Figure S4, at ratios of 1:1, 2:1, and 3:1. Overall, the 2:1 ratio showed slightly higher efficiency, which can be attributed to both the content of menthol and acetic acid. At a higher menthol-to-acetic acid molar ratio (3:1), the increased volume of the extractant-rich phase promotes dilution of the extracted BPs, thereby reducing the analytical signal. Conversely, at lower menthol-to-acetic acid ratios, the low menthol content decreases the extraction efficiency.

In addition, the presence of HBD in the NADES microextraction plays an important role as a disperser. At a higher menthol-to-acetic acid ratio (3:1), the lower acetic acid content reduces the efficiency of menthol dispersion and, consequently, BPs extraction. Thus, the 2:1 molar ratio was selected for further experiments.

### Multivariate Optimization

Table 1 shows the 2^4^ factorial design, including the factors, their levels, the individual desirability values (di), which were determined from the peak areas of each BPs, and the global desirability (Dg). The influence of the factors on the microextraction, taking the global desirability (Dg) as analytical response, can be observed graphically in the Pareto chart (Figure 3).

**Table 1:** Factorial 2^4^ Design for Screening the Factors pH, Vortex Time, Concentration of Salt in the Solution, and Volume of the NADES^a^

Runs	pH	V. T	%w/v	Vol.	d2-BP	d4-BP	d2,6-DBP	d2,4-DBP	d2,4,6-TBP	D.g (average)
1	(1)	(1)	(1)	(1)	0.5683	0.6693	0.1242	0.5037	0.0803	0.2860
	8.00	60	10	200	0.5726	0.6381	0.1313	0.4955	0.1319	0.3157
2	(−1)	(1)	(1)	(1)	0.7088	0.5419	0.6182	0.6377	0.4107	0.5738
	4.00	60	10	200	0.7690	0.6383	0.6915	0.7189	0.4859	0.6528
3	(1)	(−1)	(1)	(1)	0.2737	0.5003	0.0362	0.3886	0.0024	0.0857
	8.00	30	10	200	0.3114	0.5275	0.0311	0.3627	0.0071	0.1058
4	(−1)	(−1)	(1)	(1)	0.6431	0.4690	0.5058	0.0797	0.3239	0.3305
	4.00	30	10	200	0.6386	0.5390	0.5300	0.0850	0.3239	0.3469
5	(1)	(1)	(−1)	(1)	0.2565	0.4830	0.0242	0.2667	0.0083	0.0922
	8.00	60	0	200	0.2605	0.5270	0.0200	0.2590	0.0091	0.0918
6	(−1)	(1)	(−1)	(1)	0.5254	0.5224	0.5712	0.5589	0.4101	0.5142
	4.00	60	0	200	0.5327	0.5137	0.6287	0.5737	0.4203	0.5292
7	(1)	(−1)	(−1)	(1)	0.2649	0.5528	0.0253	0.2688	0.0000	0.0000
	8.00	30	0	200	0.2509	0.5059	0.0198	0.2674	0.0007	0.0545
8	(−1)	(−1)	(−1)	(1)	0.5217	0.5289	0.5420	0.5245	0.4193	0.5051
	4.00	30	0	200	0.5178	0.5009	0.5685	0.5337	0.4487	0.5124
9	(1)	(1)	(1)	(−1)	0.1281	0.5019	0.0000	0.2790	0.0072	0.0000
	8.00	60	10	100	0.1629	0.4721	0.0042	0.2763	0.0062	0.0562
10	(−1)	(1)	(1)	(−1)	1.0000	1.0000	0.8633	0.8537	1.0000	0.9408
	4.00	60	10	100	0.9693	0.9743	1.000	1.0000	0.6876	0.9173
11	(1)	(−1)	(1)	(−1)	0.5992	0.7774	0.2012	0.3986	0.1507	0.3549
	8.00	30	10	100	0.5721	0.8295	0.1543	0.3952	0.1392	0.3319
12	(−1)	(−1)	(1)	(−1)	0.6925	0.6969	0.7753	0.6902	0.3816	0.6291
	4.00	30	10	100	0.6843	0.6232	0.7688	0.6802	0.3739	0.6084
13	(1)	(1)	(−1)	(−1)	0.2445	0.6057	0.0165	0.2550	0.0039	0.0754
	8.00	60	0	100	0.2670	0.5990	0.0186	0.2399	0.0019	0.0674
14	(−1)	(1)	(−1)	(−1)	0.2938	0.3166	0.6117	0.5851	0.4530	0.4322
	4.00	60	0	100	0.2996	0.3805	0.6113	0.5874	0.4712	0.4540
15	(1)	(−1)	(−1)	(−1)	0.2347	0.5922	0.0339	0.1524	0.0011	0.0603
	8.00	30	0	100	0.2248	0.5153	0.0238	0.1584	0.0014	0.0573
16	(−1)	(−1)	(−1)	(−1)	0.0000	0.0000	0.0664	0.0000	0.0394	0.0000
	4.00	30	0	100	0.0107	0.0012	0.0611	0.0113	0.0398	0.0128

a The values in parentheses represent the coded values of the experimental design. Experiments carried out in duplicate. D: individual desirability; Dg: global desirability; V.T.: vortex time; %w/v: concentration of salt in the solution; Vol: volume of the NADES.

**Figure 3:**
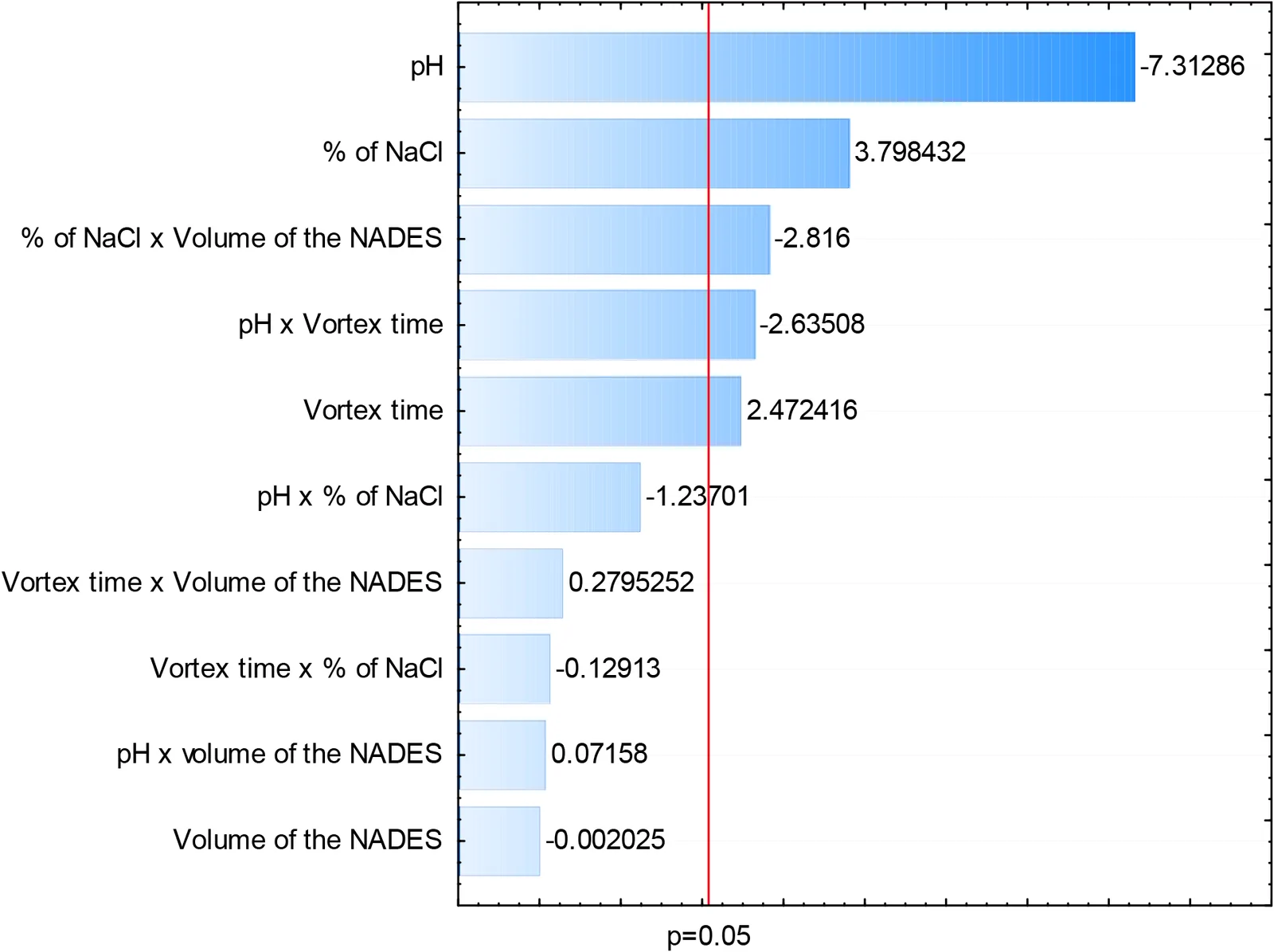
Pareto chart obtained from the 2^4^ factorial design.

Sample pH, % of NaCl, and vortex time played important roles in the microextraction procedure. The negative effect (−7.31) of sample pH indicates that, within the experimental domain (4.00 – 8.00), BPs preconcentration is favored under more acidic conditions. Under these conditions, BPs are predominantly present in their molecular form due to their p*K*_a_ values (Table S1), which consequently leads to higher extraction efficiency.^47,48^

The positive effect (3.79) of % of NaCl is attributed to the salting-out effect, in which the water activity is decreased through a reduced number of available hydrogen bonding sites, which causes a consequent increase in the coacervation process,^49–51^ reducing the solubility of BPs and enhancing their extraction in the NADES solvent.

Vortex-assisted stirring time also showed a positive effect (2.47), as it promotes the dispersion of NADES in the solution and consequently enhances analyte preconcentration. The volume of NADES within the experimental domain (100–200 μL) did not present a statistical influence in microextraction. Thus, 100 μL of NADES was chosen as the best condition.

The final optimization of sample pH, % of NaCl, and vortex time was carried out using the Doehlert experimental design (Table 2), setting the NADES volume at 100 μL.

**Table 2:** Doehlert Design for Final Optimization of Concentration of Salt in the Solution (%w/V), pH, and Vortex Time of the LLME-NADES Procedure^a^

Runs	%w/v	pH	Vortex Time	d2-BP	d4-BP	d2,6-DBP	d2,4-DBP	d2,4,6-TBP	D.g
1	(0) 11	(0) 5.00	(0) 105	1	0.9337	1	1	0.6475	0.9042
1	(0) 11	(0) 5.00	(0) 105	0.9563	1	0.9008	0.9210	0.6854	0.8853
1	(0) 11	(0) 5.00	(0) 105	0.9717	0.9602	0.9867	0.8386	0.6345	0.8670
2	(1) 20	(0) 5.00	(0) 105	0.6691	0.4741	0.5193	0.4888	0.2937	0.4729
3	(0.5) 15.5	(0.866) 8.00	(0) 105	0.6881	0.5542	0.7712	0.2544	0.3873	0.4925
4	(0.5) 15.5	(0.289) 4.00	(0.817) 180	0.8705	0.6510	0.8539	0.5579	0.3512	0.6243
5	(−1) 2	(0) 5.00	(0) 105	0	0	0	0	0.0140	0
6	(−0.5) 6.5	(−0.866) 2.00	(0) 105	0.7141	0.6598	0.6387	0.5892	1	0.7075
7	(−0.5) 6.5	(−0.289) 4.00	(−0.817) 30	0.5355	0.4515	0.4792	0.3432	0.2854	0.4083
8	(0.5) 15.5	(−0.866) 2.00	(0) 105	0.7654	0.5026	0.6074	0.5787	0.5522	0.5951
9	(0.5) 15.5	(−0.289) 4.00	(−0.817) 30	0.8238	0.5574	0.6582	0.7563	0.3163	0.5913
10	(−0.5) 6.5	(0.866) 8.00	(0) 105	0.6020	0.4712	0.7499	0.4143	0.0712	0.3627
11	(0) 11	(0.577) 7.00	(−0.817) 30	0.2629	0.3513	0.3203	0.0176	0	0
12	(−0.5) 6.5	(0.289) 6.00	(0.817) 180	0.4834	0.4965	0.5154	0.7363	0.3505	0.5021
13	(0) 11	(−0.577) 3.00	(0.817) 180	0.8109	0.7242	0.7193	0.3589	0.6391	0.6270

a The values in parentheses represent the coded values of experimental design. D: individual desirability; Dg: global desirability.

The quadratic model (eq 3), obtained from the Doehlert design, was validated by analysis of variance ANOVA (Table 3).

3 $$D_{g}=-0.3366_{\pm 0.1693}+0.1880_{\pm 0.0137}\%salt^{*}-0.0077_{\pm 0.0004}\%salt^{2*}-0.0805_{\pm 0.0414}pH^{*}-0.0186_{\pm 0.0030}pH^{2*}-0.0056_{\pm 0.0013}vortex\;time^{*}-0.0001_{\pm 0.00004}vortex\;time^{2*}+0.0047_{\pm 0.0014}\%salt\times pH-0.0002_{\pm 0.00006}\%salt\times vortex\;time+0.0015_{\pm 0.0001}pH\times vortex\;time^{*}$$

**Table 3:** Analytical Characteristics of the Proposed Method for the Determination of BPs^a^

Analyte	Linear range (μg L^–1^)	Linear equation	EF	LOD (μg L^–1^)	LOQ (μg L^–1^)
2-BP	0.49–100.0	Area= 2847.44 ± 27.91 [2-BP] + 5191.16 ± 63.35	90.80	0.14	0.49
		R^2^ = 0.9991			
4-BP	1.11–100.0	Area= 1089.45 ± 26.82 [4-BP] + 1713.40 ± 70.58	102.3	0.33	1.11
		R^2^ = 0.9967			
2,6-DBP	1.47–100.0	Area= 1962.08 ± 14.30 [2,6-DBP] + 495.98 ± 31.26	90.25	0.44	1.47
		R^2^ = 0.9977			
2,4-DBP	0.90–100.0	Area= 2136.42 ± 21.27 [2,4-DBP] – 3649.07 ± 49.81	110.35	0.27	0.90
		R^2^ = 0.9981			
2,4,6-TBP	0.81–100.0	Area= 1692.41 ± 31.31 [2,4,6-TBP] + 1781.85 ± 67.03	120.80	0.24	0.81
		R^2^ = 0.9987			

a EF: Enrichment factor; LOD: Limit of detection; LOQ: Limit of quantification.

The values marked with an asterisk (*) are statistically significant (*p* < 0.05). Higher determination coefficients, *R* ^2^ = 0.98928 and adjusted *R* ^2^ = 0.9399 were obtained. Furthermore, ANOVA, no lack-of-fit was observed, since the MSl_ack of fit_/MS_pure error_ ratio of 12.42 was lower than the critical F-value (F _3,2,95%_= 19.16). The response surfaces obtained from the validated quadratic model are depicted in Figure 4.

**Figure 4:**
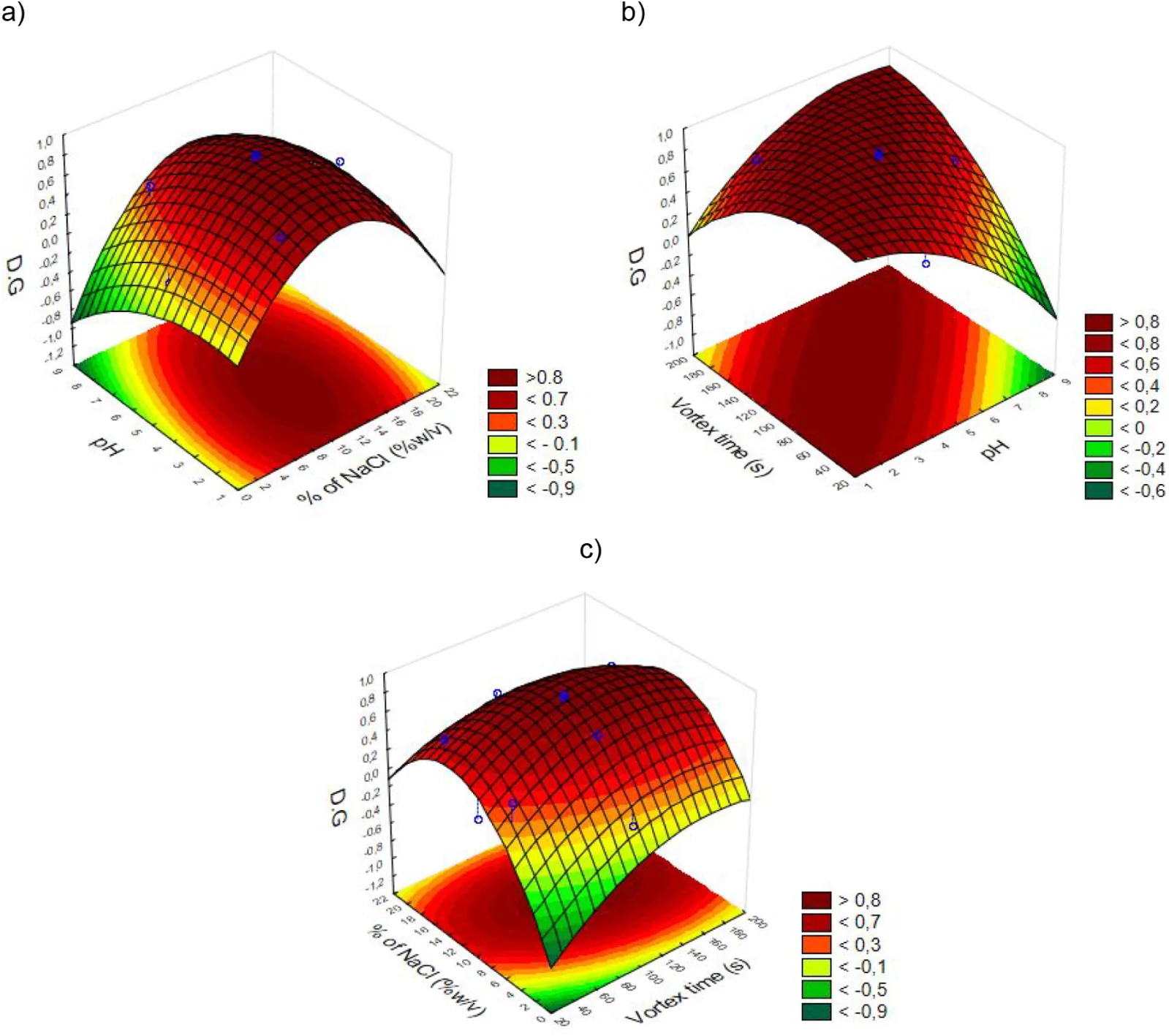
Response surface for pH× % of NaCl (%w/v) (a), Vortex time (s) × pH (b), % of NaCl (%w/v) × Vortex time (s) (c). Conditions: NaCl and 100 μL of the NADES M:AA (2:1 molar ratio).

The respective critical points for pH, concentration of the salt in solution (%w/v), and time of the vortex were found to be 3.92, 11.56%, and 108 s. Under these optimized conditions, a significant improvement in BPs detectability was observed, as shown in Figure 5.

**Figure 5:**
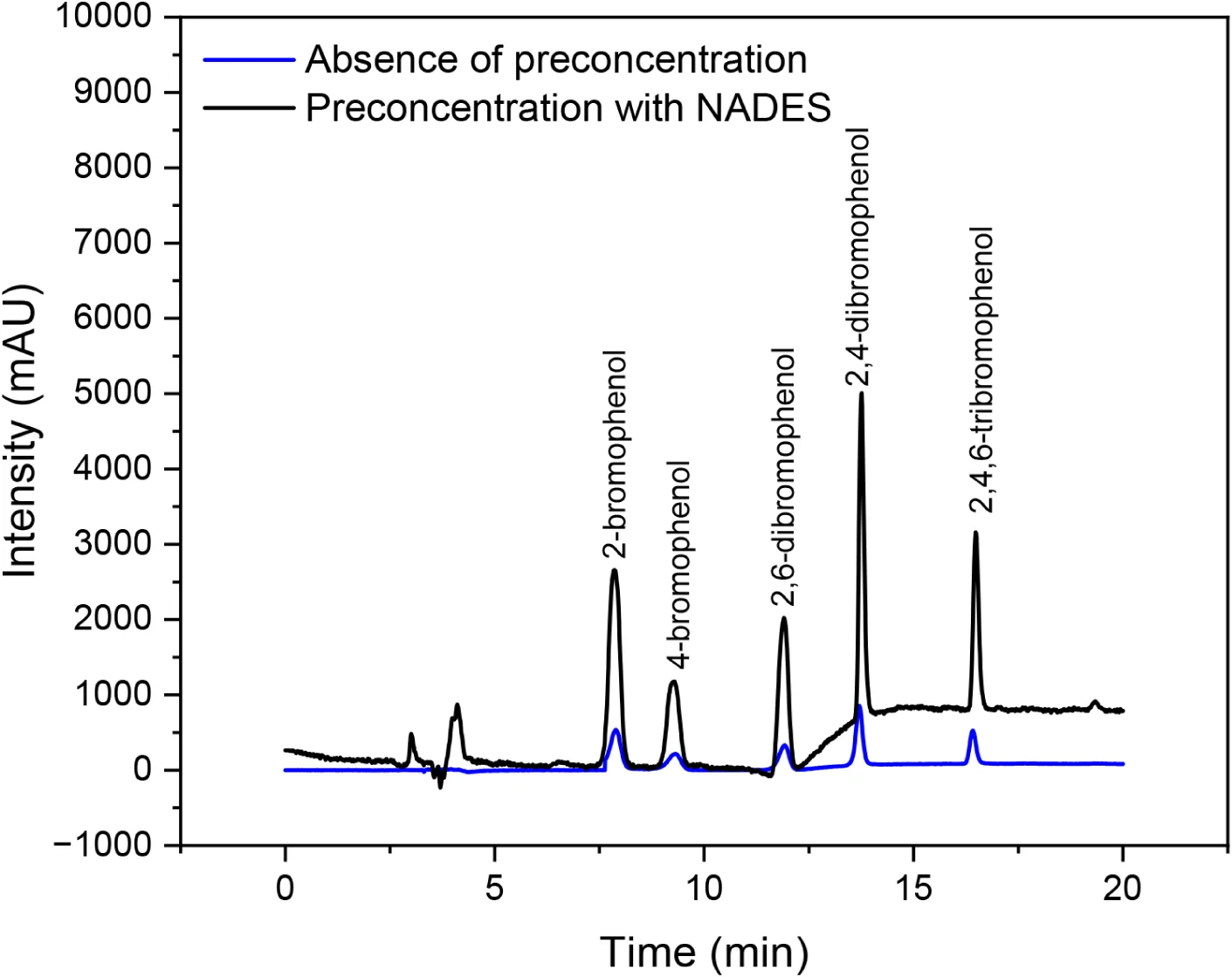
Chromatograms 2-BP, 4-BP, 2,6-DBP, 2,4-DBP, and 2,4,6-TBP at 500 μg L^–1^ without preconcentration (blue line) and after NADES microextraction (black line) by preconcentrating 40.0 mL of solution. Conditions: 20 μg L^–1^ of the 2-BP, 4-BP, 2,6-DBP, 2,4-DBP, and 2,4,6-TBP, pH 3.92 (Buffer BR 0.01 mol L^–1^), 11.6% NaCl, 100 μL of NADES, and 108 s of vortex time.

### Analytical Parameters and Applicability of the Method

Table 3 and Table S3 depict the range of linear calibration curve, limits of detection (LOD) and quantification (LOQ), enrichment factor (EF), the intraday precision, and intermediate precision.

The linear regressions obtained were statistically evaluated by analysis of variance (ANOVA) at a 95% confidence level. The MS_Regression_/MS_residual_ ratios were much higher than the F_tab_ value (3.39), indicating the statistical significance of the models. Higher EF values were obtained, ranging from 90.8 to 120.80. It was observed that the higher LogK_ow_, the higher EF. The intraday and intermediate precision (Table 3S) ranged from 1.14 to 4.70%, indicating the satisfactory precision of the proposed method.^52^

Analyses of water samples (lake and river), sawdust powder, and fish meal were carried out to assess the feasibility of the proposed method. The results are shown in Table 4.

**Table 4:** Analysis of the Samples and Recovery Values (*N* = 3)^a^

Analyte	Concentration added				Concentration found				Recovery (%)		
**Sample location: Igapo Lake (Londrina city) -** **μg L** ^**–1**^											
2-BP	0.0	5.0	20.0	75.0	<LOQ	5.8 ± 0.6	19.8 ± 1.2	76.1 ± 1.3	116	99	101
4-BP	0.0	5.0	20.0	75.0	<LOQ	5.4 ± 0.9	18.2 ± 0.7	74.6 ± 1.1	107	90	99
2,6-DBP	0.0	5.0	20.0	75.0	<LOQ	4.5 ± 0.3	19.7 ± 2.3	73.9 ± 1.2	90	98	98
2,4-DBP	0.0	5.0	20.0	75.0	<LOQ	5.9 ± 1.2	19.1 ± 1.8	70.9 ± 2.6	119	95	94
2,4,6-TBP	0.0	5.0	20.0	75.0	<LOQ	4.6 ± 0.9	21.5 ± 0.4	73.9 ± 1.1	91	107	98
**Sample location: Bandeira do Norte River (Arapongas city) -** **μg L** ^**–1**^											
2-BP	0.0	5.0	20.0	75.0	<LOQ	5.9 ± 0.2	20.8 ± 1.5	74.8 ± 2.5	118	104	99
4-BP	0.0	5.0	20.0	75.0	<LOQ	5.2 ± 0.3	18.5 ± 0.7	81.3 ± 2.1	105	92	108
2,6-DBP	0.0	5.0	20.0	75.0	<LOQ	4.5 ± 0.1	20.3 ± 0.6	71.3 ± 1.7	90	101	95
2,4-DBP	0.0	5.0	20.0	75.0	<LOQ	5.9 ± .0.2	19.2 ± 0.4	69.6 ± 0.6	118	95	92
2,4,6-TBP	0.0	5.0	20.0	75.0	<LOQ	5.3 ± 0.5	19.9 ± 2.2	72.9 ± 1.7	105	99	97
**Sawdust powder -** mg kg^**–** **1**^											
2-BP	0.0	10.0	40.0	150.0	<LOQ	10.6 ± 2.1	38.2 ± 3.7	146.7 ± 5.2	105	95	97
4-BP	0.0	10.0	40.0	150.0	<LOQ	9.8 ± 1.3	36.5 ± 0.8	151.7 ± 0.9	97	91	101
2,6-DBP	0.0	10.0	40.0	150.0	<LOQ	9.0 ± 1.0	39.1 ± 3.3	147.9 ± 0.9	90	97	98
2,4-DBP	0.0	10.0	40.0	150.0	<LOQ	11.8 ± 0.2	39.3 ± 1.6	142.4 ± 1.4	118	98	94
2,4,6-TBP	0.0	10.0	40.0	150.0	<LOQ	9.0 ± 0.5	42.8 ± 1.1	146.9 ± 0.8	90	107	97
**Fish Meal -** mg kg**^–^** ^**1**^											
2-BP	0.0	10.0	40.0	150.0	1.6 ± 0.5	12.9 ± 0.5	45.0 ± 1.6	172.6 ± 1.8	113	108	114
4-BP	0.0	10.0	40.0	150.0	36.7 ± 1.3	48.4 ± 0.5	82.1 ± .1.5	207.7 ± 2.7	116	113	113
2,6-DBP	0.0	10.0	40.0	150.0	4.9 ± 0.2	15.4 ± 0.3	47.4 ± 1.4	172.9 ± 1.0	104	106	112
2,4-DBP	0.0	10.0	40.0	150.0	<LOQ	11.8 ± 0.6	42.4 ± 1.6	154.9 ± 4.4	118	106	103
2,4,6-TBP	0.0	10.0	40.0	150.0	<LOQ	11.4 ± 1.5	41.9 ± 1.7	152.4 ± 3.9	113	104	101

a <LOQ: Lower than the limit of quantification.

One should note that recovery values are consistently satisfactory (90% to 119%), within the acceptable range established by the AOAC guideline.^52^ These values were obtained using external calibration curves (Figure S6), indicating the absence of significant matrix effects even in samples with different and complex matrices.

Figure 6 depicts chromatograms of spiked samples, including river and lake water, sawdust powder, and fish meal. No BPs were detected in the nonspiked water (river and lake) and sawdust powder samples; however, clear and well-defined peaks appeared at spiked concentrations of 5.0, 20.0, and 75.0 μg L^–1^ in water (lake and river samples) and 10.0, 40.0, and 150.0 mg kg^–1^ in sawdust powder. These results corroborate the method’s effectiveness in detecting BPs without interference and confirm the absence of significant matrix effects.

**Figure 6:**
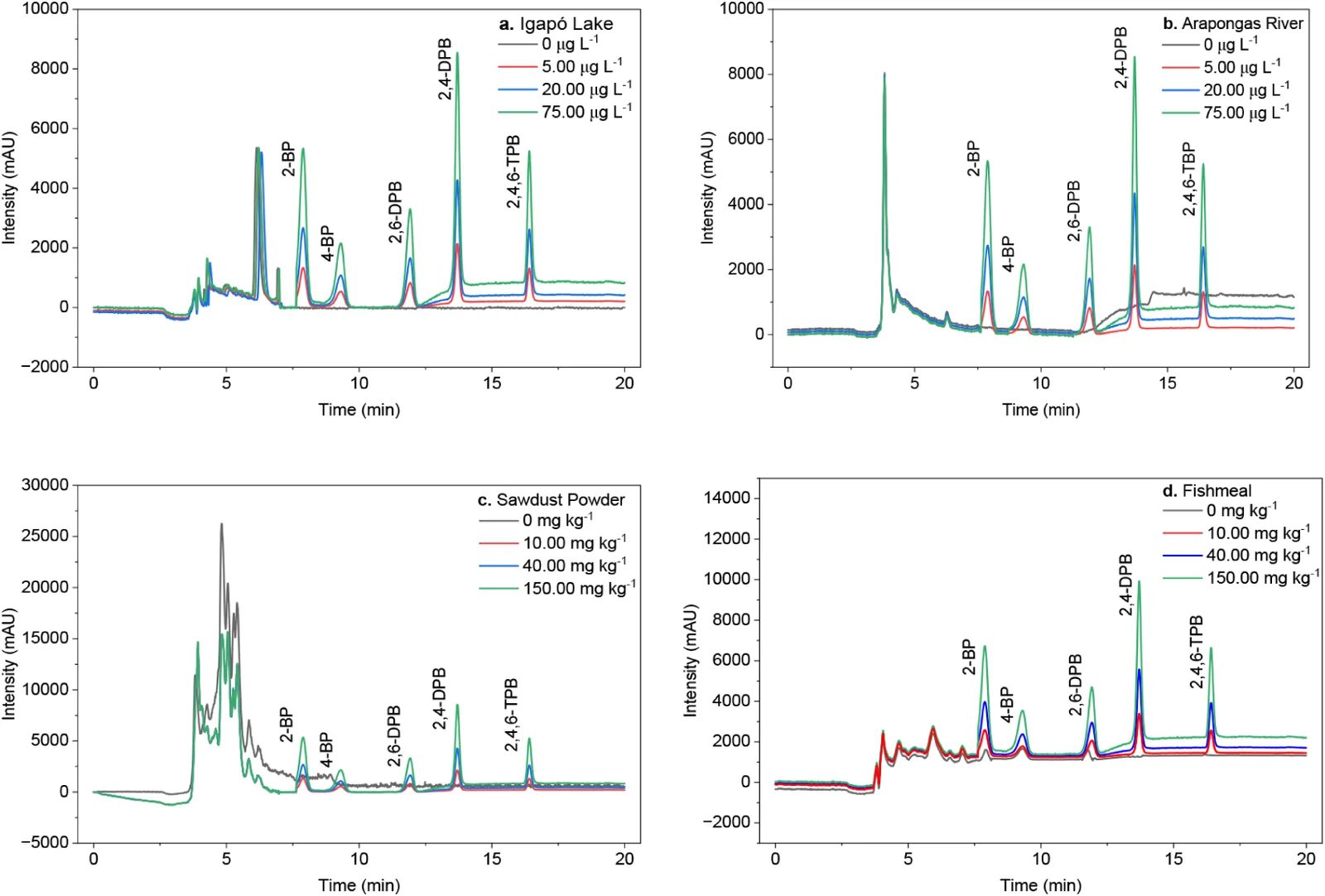
HPLC-DAD chromatograms obtained from the addition/recoveries test for (a) Igapó Lake, (b) Arapongas River, (c) Sawdust Powder, and (d) Fishmeal samples.

For nonspiked fish meal samples, well-defined peaks at the same retention time as 2-BP, 4-BP, and 2,6-DBP (Figure 4d) were observed, indicating the presence of BPs at concentrations of 1.6, 36.7, and 4.9 mg kg^–1^, respectively. HPLC-MS analysis was performed to confirm the presence of BPs. The mass spectra of the sample present the [M – H]^+^ ion at 174.000, which corresponds to the *m*/*z* of the 2-BP and 4-BP, in their respective retention times, and present the [M – H]^+^ ion at 252.000, which corresponds to the *m*/*z* of the 2,6-DBP.^47,53^ The mass spectra are shown in Figure 7S. The occurrence of these analytes in seafood samples has been previously reported in the literature.^12^

Chi and co-workers^1^ reported on the presence of the BPs in environmental samples. 4-BP, 2,4-DBP, and 2,4,6-TPB were determined in factory discharge sewage from Haidian district, Beijing, in the concentrations of 0.67 μg L^–1^, 16.93 μg L^–1^, and 70.62 μg L^–1^, respectively. All the BPs in the wastewater sample might be attributed to industrial production, as byproducts of TBBPA and other brominated flame retardants, which are the factory’s major products. These findings confirm the suitability of the method for detecting BPs in environmental samples based on the analytical parameters obtained.

Compared with previously reported studies (Table 5), the proposed method achieved a substantially higher EF (90.25–120.8), which consequently contributes to lower LOD and LOQ. These results demonstrate that the method provides greater analytical sensitivity than other approaches employing HPLC-UV for the detection of BPs.^3,17^ In addition, the method is more environmentally sustainable because it uses NADES rather than conventional organic solvents. Furthermore, the analytical technique employed entails lower operational costs than methods reported in the literature.

**Table 5:** Comparison of Literature Methods and the Present Method for BPs Determination in Different Matrices^a^

Analytes	Sample preparation	Technique	Sample	Solvent/volume of extraction	EF (fold)	Linear range (μg L^–1^)	LOQ(μg L^–1^)	LOD(μg L^–1^)	Reference
2-BP	Hydrodistillation-solvent extraction	RP-HPLC-UV	Fish	2 mL of pentane/diethyl ether (6:4, v/v)	–	200–1000	424	127	^3^
4-BP							596	179	
2,4-DBP							297	89	
2,6-DBP							898	269	
2,4,6-TBP							774	232	
4-BP	LLE and aqueous derivatization with acetic anhydride	GC-MS	Synthetic and human urine	0.5 mL of hexane	–	–	0.027	0.008	^10^
2,4-DBP							0.088	0.026	
2,6-DBP							0.085	0.025	
2,4,6-TBP							0.026	0.007	
2,4,6-TBP	LLE and derivatization with BSTFA	GC-MS	Wood	0.1 mL of toluene	–	0.5–50	–	1.5	^13^
2-BP	Hydrodistillation-solvent extraction	HPLC-UV	Fish	2 mL of pentane/diethyl ether (6:4, v/v)	–	200–1000	–	–	^56^
4-BP									
2,4-DBP									
2,6-DBP									
2,4,6-TBP									
2-BP	LLE using NADES	HPLC-DAD	River and lake water, Sawdust Powder, and Fish Meal	0.1 mL of the NADES M: AA (2:1 molar ratio)	90.80	0.49–100.0	0.49	0.14	This study
4-BP					102.3	1.11–100.0	1.11	0.33	
2,4-DBP					90.25	1.47–100.0	1.47	0.44	
2,6-DBP					110.35	0.90–100.0	0.90	0.27	
2,4,6-TBP					120.80	0.81–100.0	0.81	0.24	

a EF: enrichment factor; RP-HPLC-UV: reverse-phase high-performance liquid chromatography with ultraviolet detection; LLE: liquid–liquid extraction; GC-MS: gas chromatography–mass spectrometry; BSTFA: N,O-bis­(trimethylsilyl)­trifluoroacetamide; HPLC-UV-EC: high-performance liquid chromatography with ultraviolet and electrochemical detectors.

### Assessment of the Method Greenness Profile

The greenness of the microextraction method was evaluated using the Blue Applicability grade Index (BAGI)^57^ and the AGREE tool,^58^ which assesses the practicality of analytical procedures. The results are illustrated in Table S4.

According to the BAGI assessment, a score of 65.0 falls within the acceptable range of applicability. Scores below 25 indicate limited applicability, whereas a score of 100 represents excellent applicability. This score was achieved because the method consists of a quantitative multielement analysis performed using a semiautomated HPLC-DAD technique, combined with a miniaturized sample-preparation (100 μL of the NADES) procedure that also serves as a preconcentration system.

The AGREE score (0.66) indicates that the sampling process is straightforward (highlighted in green), involving only stirring and centrifugation prior to instrumental analysis, and does not require derivatization. However, its main limitation is associated with the offline nature of the analysis and the use of an HPLC-DAD system, whose energy consumption was estimated at approximately 0.18 kWh per analysis. These factors negatively influence the overall greenness assessment despite the simplicity of the sample preparation procedure.

Overall, the microextraction method using NADES requires minimal sample and solvent volume, resulting in very low waste generation and minimal toxicity and risk to the user. Furthermore, compared with HPLC-UV based methods for BP determination,^3,54,55^ the proposed method exhibits superior sensitivity and is more environmentally friendly.

## Conclusion

The NADES-based method developed for BPs determination is presented as an alternative, environmentally friendly approach for the analysis of water, sawdust powder, and fish meal samples, especially considering the limited number of studies on BPs determination by HPLC-DAD.

Among the prepared NADESs, the combination of menthol and acetic acid (2:1 molar ratio) showed slightly higher efficiency than menthol:formic acid (2:1 molar ratio) and menthol:decanoic acid (2:1 molar ratio).

The method was successfully applied to the determination of BPs in real samples using external calibration, clearly demonstrating the absence of matrix effects. The LOQ achieved is adequate for the screening and occurrence assessment of BPs in river and lake water, sawdust powder, and fish meal samples, in line with concentrations reported in the literature.

Apart from its environmentally friendly characteristics, as evaluated by the BAGI and AGREE tools, the proposed method stands out for its simplicity, low cost, and short microextraction time (180 s), which is considerably shorter than that of other sample pretreatment procedures.

## Supplementary Material



## Notes

Physical and chemical properties of brominated phenols; FTIR and ^1^H NMR spectra of the HBA, HBDs, and the NADES prepared at different molar ratios; effect of NADES composition and molar ratio of the NADES composition on the microextraction of BPs; analysis of variance (ANOVA) from a Doehlert design; analytical performance of the BPs preconcentration method; mass spectra of the analyzed samples; BAGI and AGREE evaluation for the BP determination methods (PDF)
