# Supplementary material for: Determination of Multi-Residue of Brominated Phenols in Water, Sawdust Powder, and Fish Meal Samples by Preconcentration with Hydrophobic Menthol-Based Natural Deep Eutectic Solvent and HPLC-DAD
Source: ACS Omega. 2026 Aug 4;11(32):47779–92. doi: 10.1021/acsomega.6c03071 (PMC13491502; doi:10.1021/acsomega.6c03071)
Supplement: Supplementary file 1 [file ao6c03071_si_001.pdf]

SUPPLEMENTARY MATERIAL

Determination of multi-residue of brominated phenols in water, sawdust powder, and fish meal samples by preconcentration with hydrophobic menthol-based natural deep eutectic solvent and HPLC-DAD

Letícia Maria Efftingª, Thifany Miyuki Terossi Makitaª, João Victor Pastorin De Carvalhoª, Mariana Gava Segatelliª, Cesar Ricardo Teixeira Tarleyª*

ªLaboratório de Desenvolvimento de Métodos Analíticos (LADEMA), Departamento de Química, Universidade Estadual de Londrina (UEL), 86057-970 Londrina-PR, Brazil;

*tarley@uel.br

Table S1. Physical and chemical properties of brominated phenols

Brominated Phenols	Structure	Molecular formula	Molecular weight (g mol^{-1})	Log K_{ow}	Density (g cm^3, 20 °C)	Boiling Point (ºC)	pK_a
2-bromophenol (2-BP)	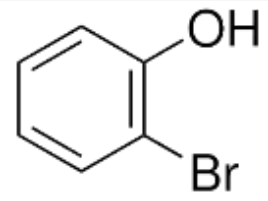	C_6H_5BrO	173.01	2.35	1.49	194.5	8.45
4-bromophenol (4-BP)	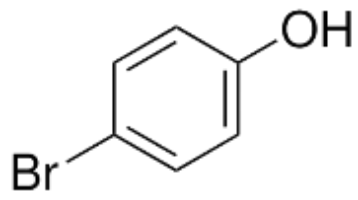	C_6H_5BrO	173.01	2.59	1.84	238	9.17
2,6-dibromophenol (2,6-DBP)	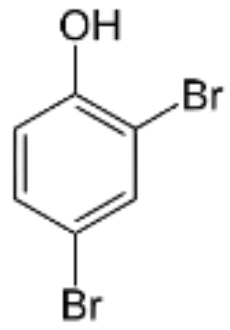	$C_6H_4Br_2O$	251.90	3.36	1.88	255	6.67
2,4-dibromophenol (2,4-DBP)	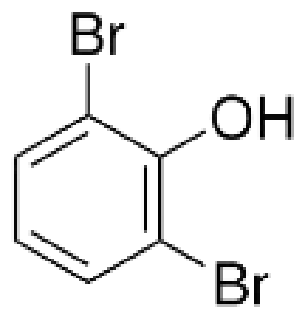	$C_6H_4Br_2O$	251.90	3.22	2.07	238.5	7.79
2,4,6-tribromophenol (2,4,6-TBP)	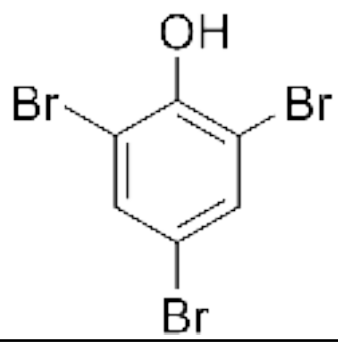	$C_6H_3Br_3O$	330.80	4.13	2.55	285	6.80

Figure 1Sa. FTIR spectra of (a) menthol, (b) acetic acid, (c) NADES M: AA (1:1 molar ratio), (d) NADES M: AA (2:1 molar ratio), and (e) NADES M: AA (3:1 molar ratio).

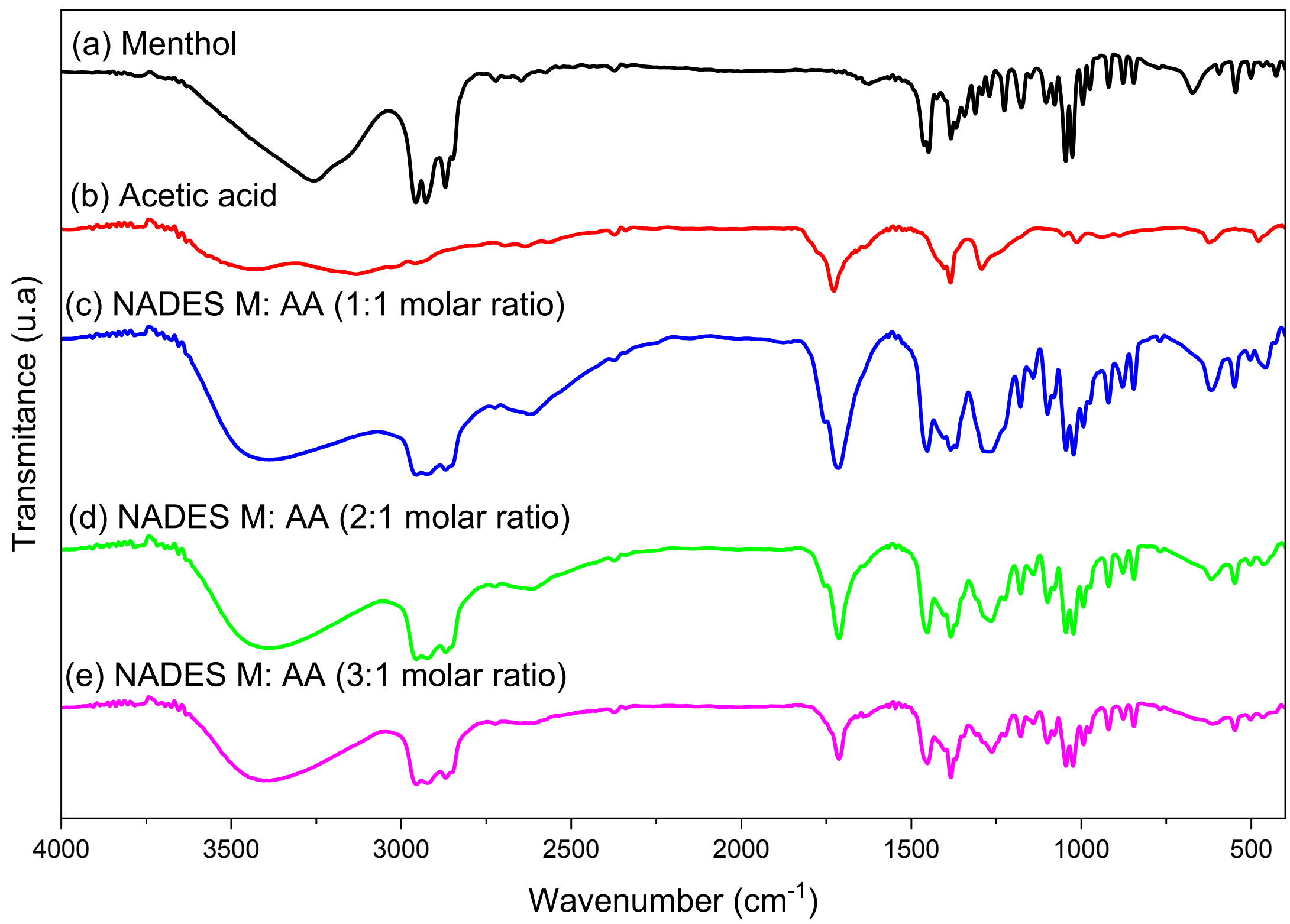

Figure 1Sb. FTIR spectra of (a) menthol, (b) formic acid, (c) NADES M: FA (2:1 molar ratio).

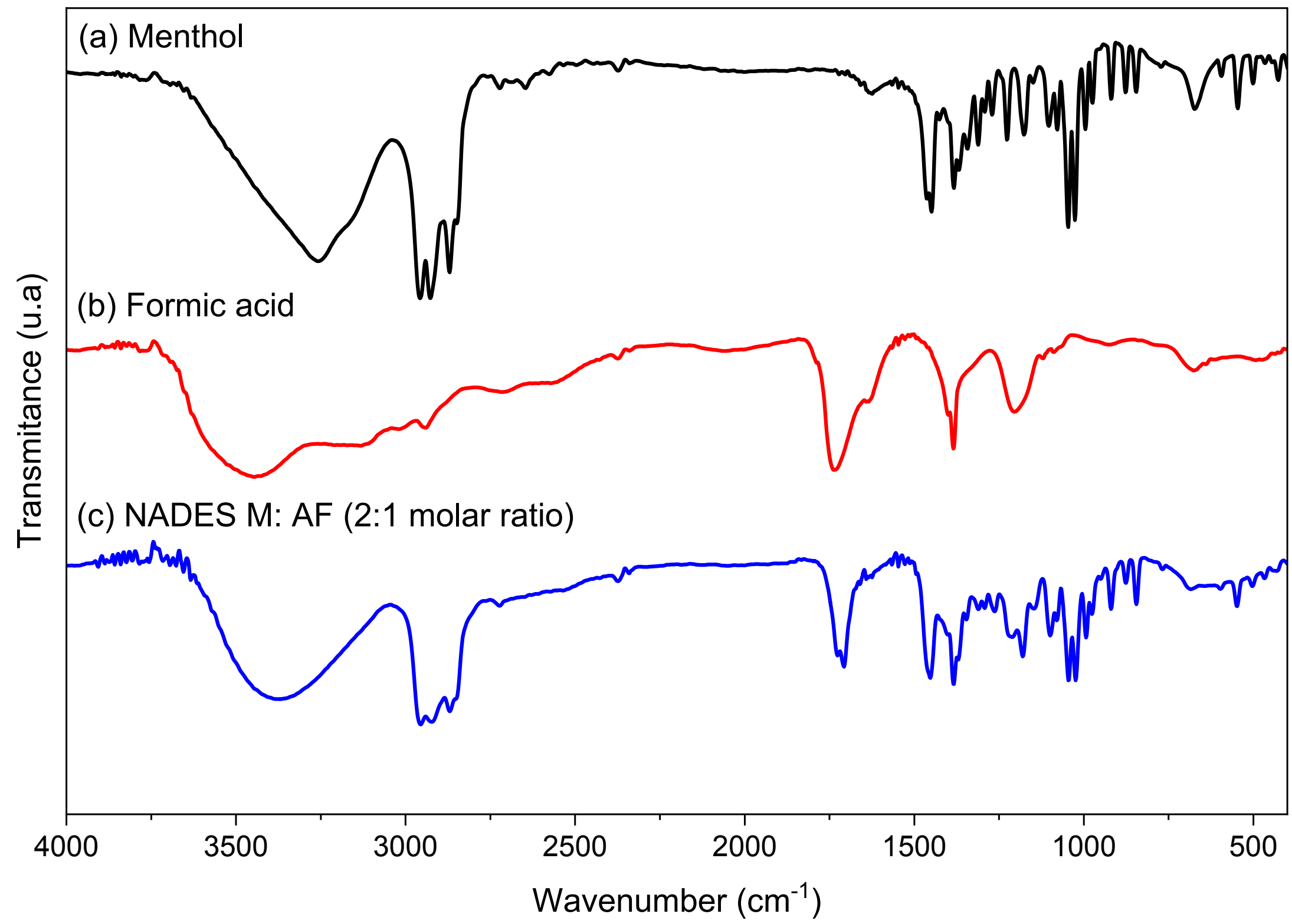

Figure 1Sc. FTIR spectra of (a) menthol, (b) formic acid, (c) NADES M: DA (2:1 molar ratio).

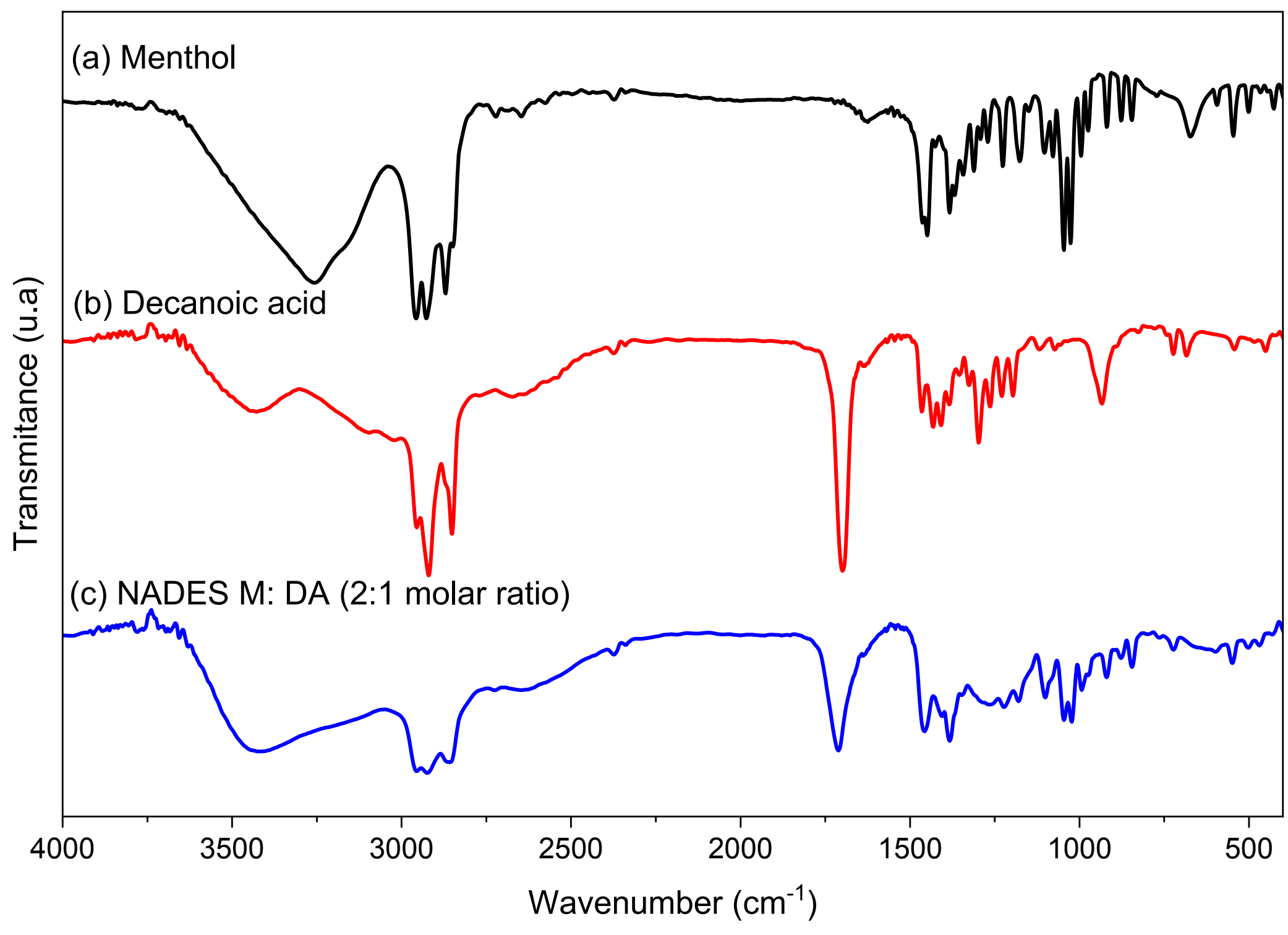

Figure 2Sa - ^{1}H NMR spectra of (a) menthol, (b) acetic acid, (c) NADES M: AA (1:1 molar ratio), (d) rich phase after microextraction with NADES M: AA (1:1 molar ratio), (e) NADES M: AA (2:1 molar ratio), (f) rich phase after microextraction with NADES M: AA (2:1 molar ratio), (g) NADES M: AA (3:1 molar ratio), and (h) rich phase after microextraction with NADES M: AA (3:1 molar ratio), under optimized conditions.

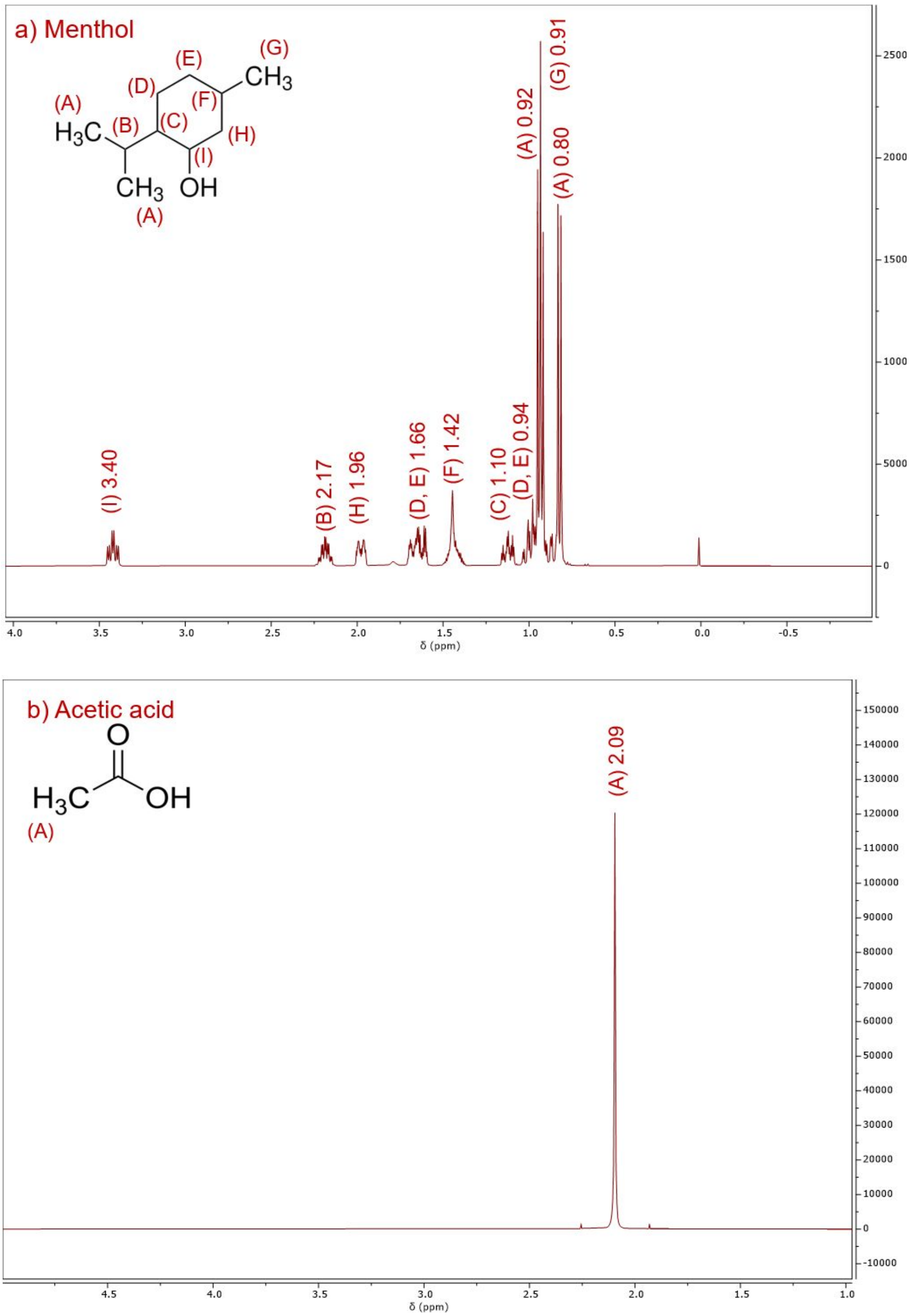

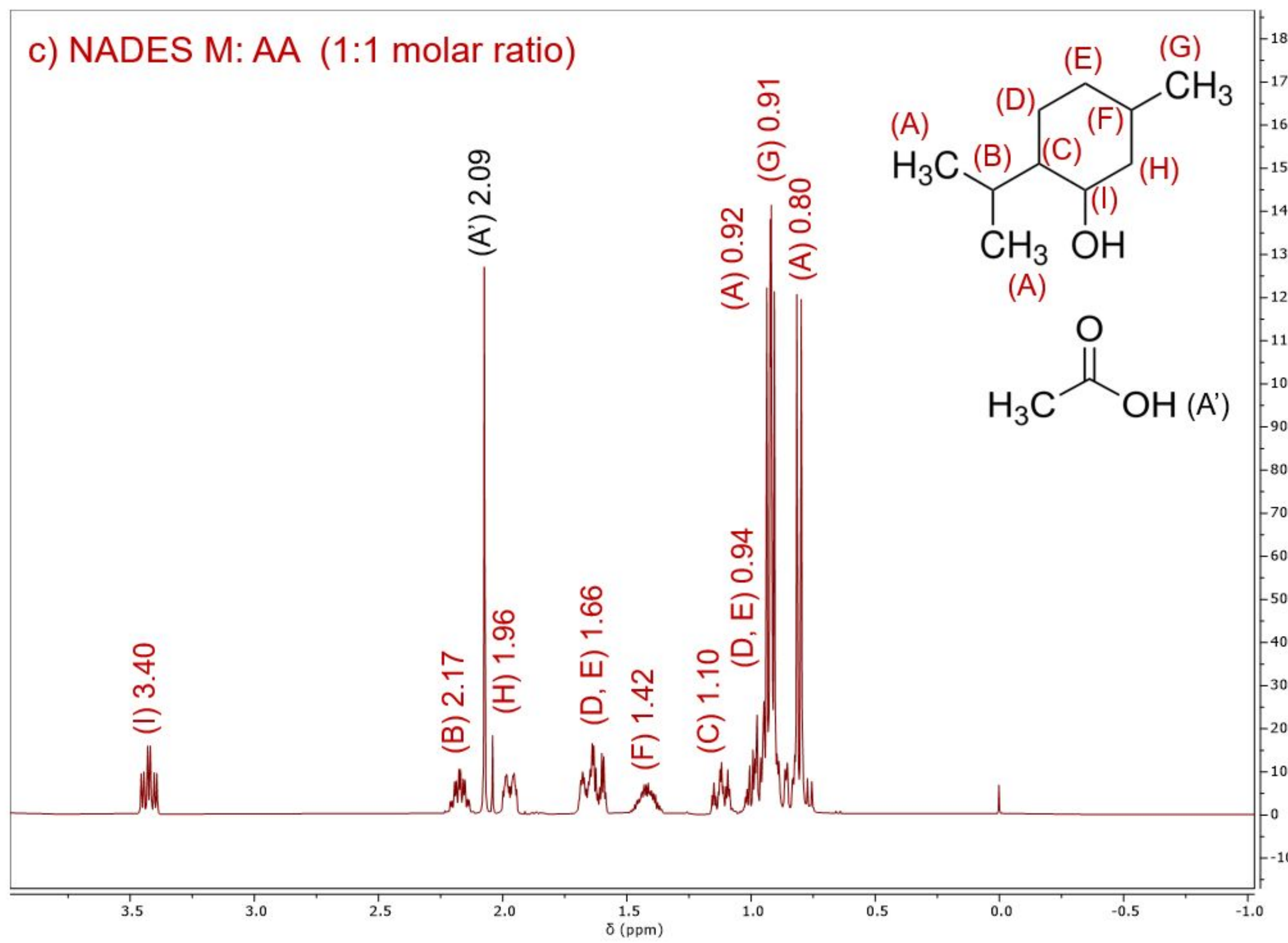
c) NADES M: AA (1:1 molar ratio)
(I) 3.40
(B) 2.17
(A') 2.09
(H) 1.96
(D, E) 1.66
(F) 1.42
(C) 1.10
(D, E) 0.94
(A) 0.92
(G) 0.91
(A) 0.80
δ (ppm)

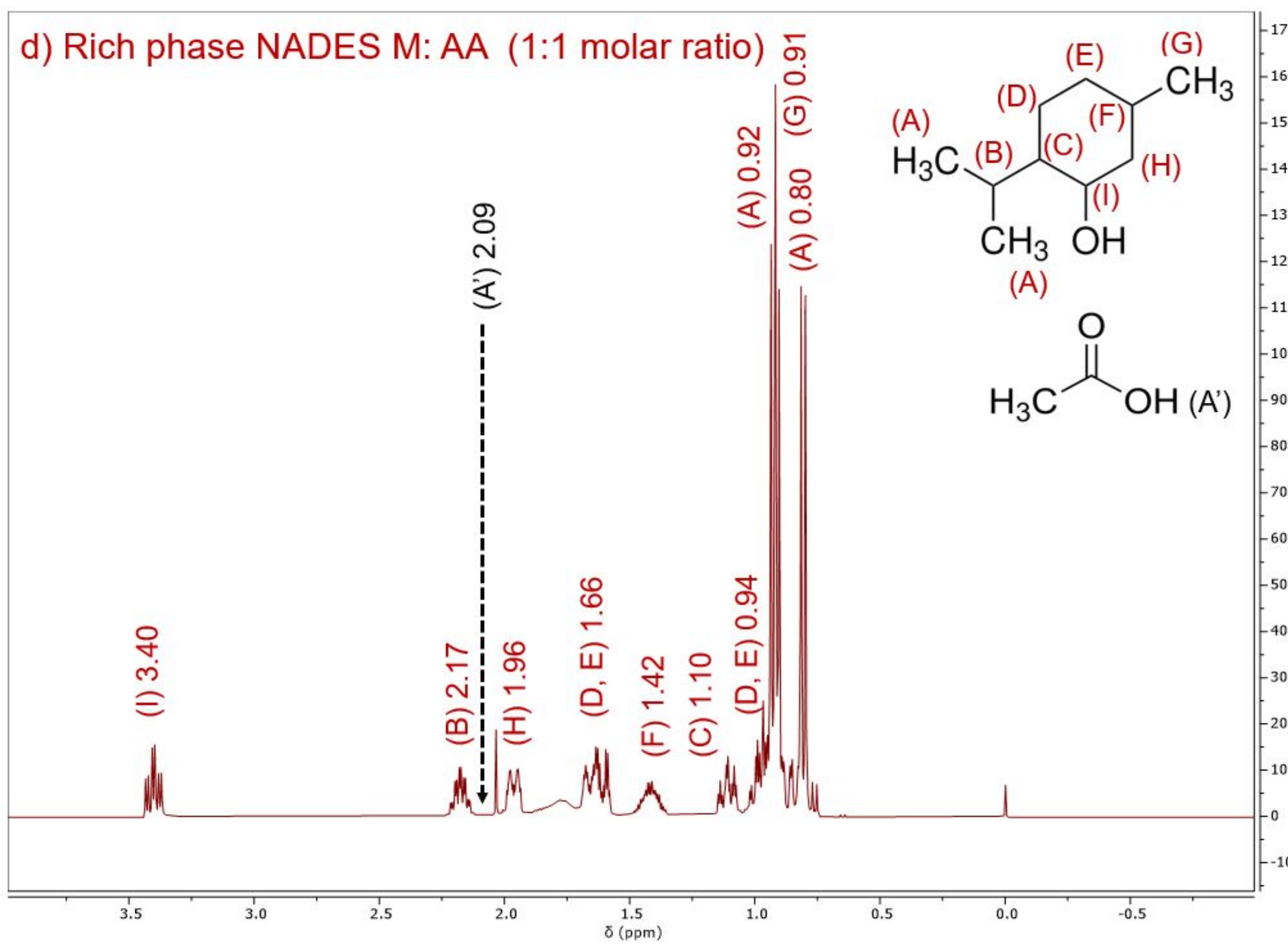
d) Rich phase NADES M: AA (1:1 molar ratio)
(I) 3.40
(B) 2.17
(A') 2.09
(H) 1.96
(D, E) 1.66
(F) 1.42
(C) 1.10
(D, E) 0.94
(A) 0.92
(G) 0.91
(A) 0.80
δ (ppm)

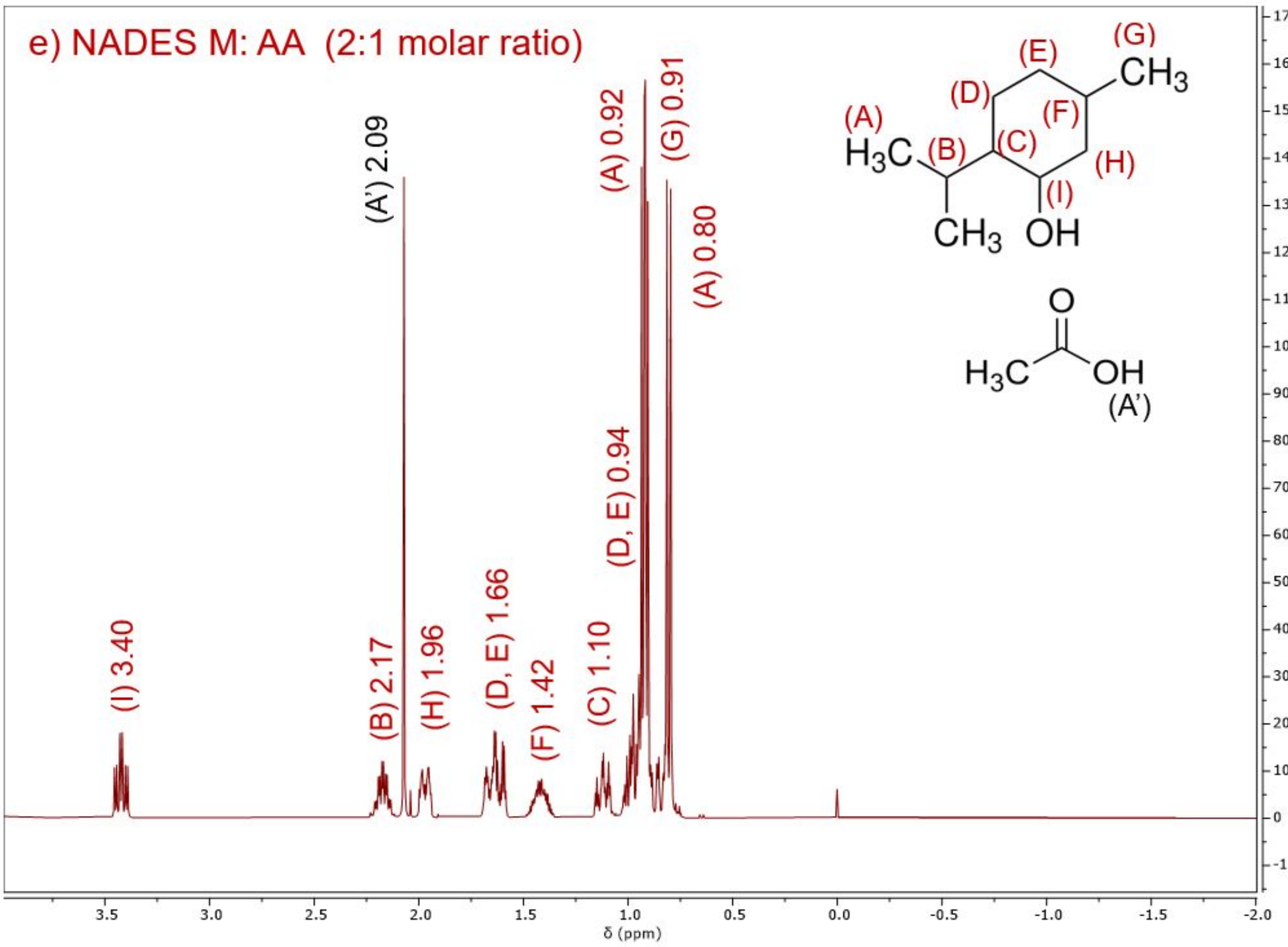
e) NADES M: AA (2:1 molar ratio)
(A') 2.09
(A) 0.92
(G) 0.91
(A) 0.80
(D, E) 0.94
(I) 3.40
(B) 2.17
(H) 1.96
(D, E) 1.66
(F) 1.42
(C) 1.10
(A)
(B)
(C)
(D)
(E)
(F)
(G)
(H)
(I)
H_3C
CH_3
CH_3
OH
O
H_3C
OH
(A')
δ (ppm)

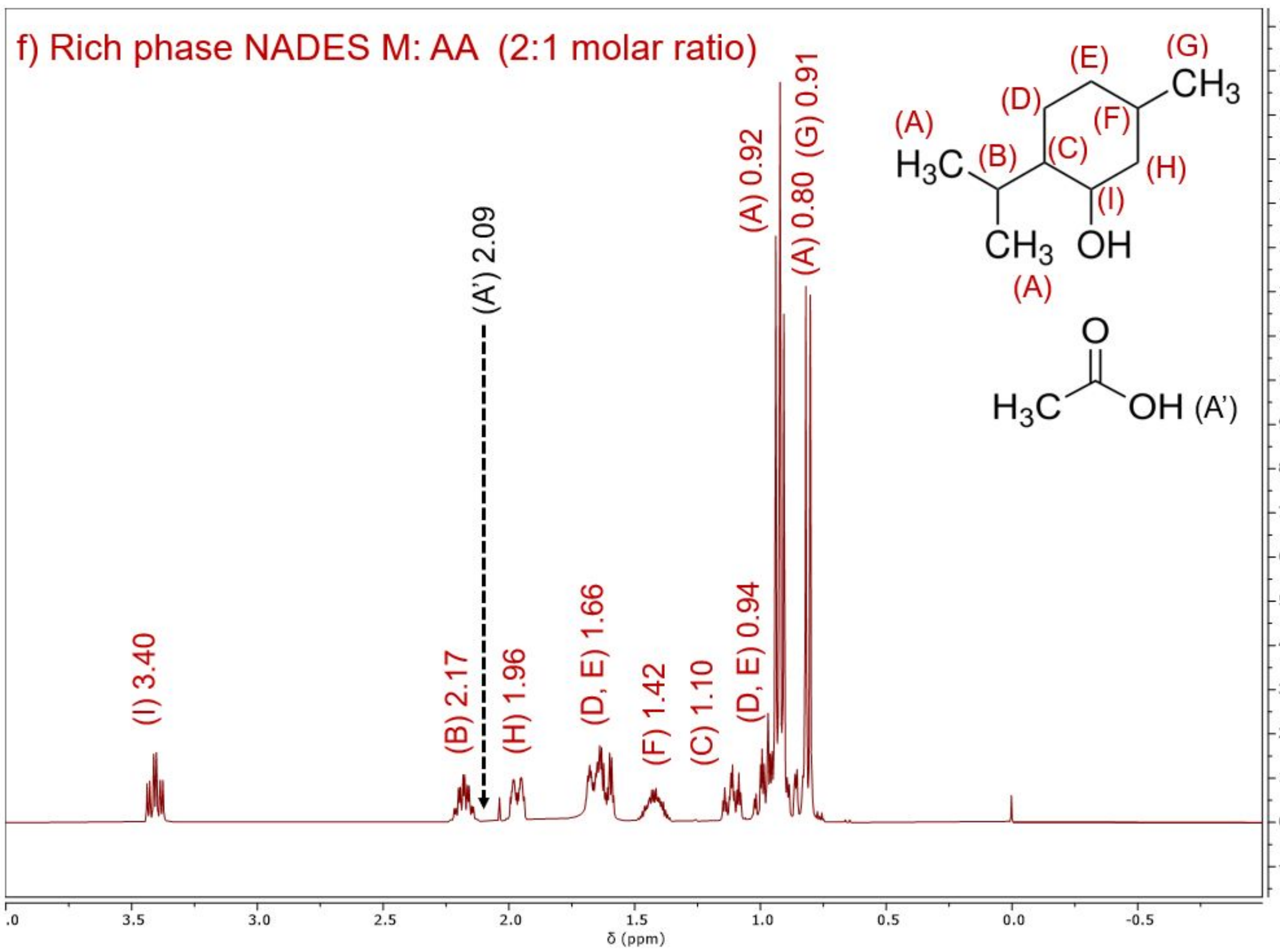
f) Rich phase NADES M: AA (2:1 molar ratio)
(A') 2.09
(A) 0.92
(A) 0.80
(G) 0.91
(I) 3.40
(B) 2.17
(H) 1.96
(D, E) 1.66
(F) 1.42
(C) 1.10
(D, E) 0.94
(A)
(B)
(C)
(D)
(E)
(F)
(G)
(H)
(I)
H_3C
CH_3
CH_3
OH
(A)
O
H_3C
OH (A')
δ (ppm)

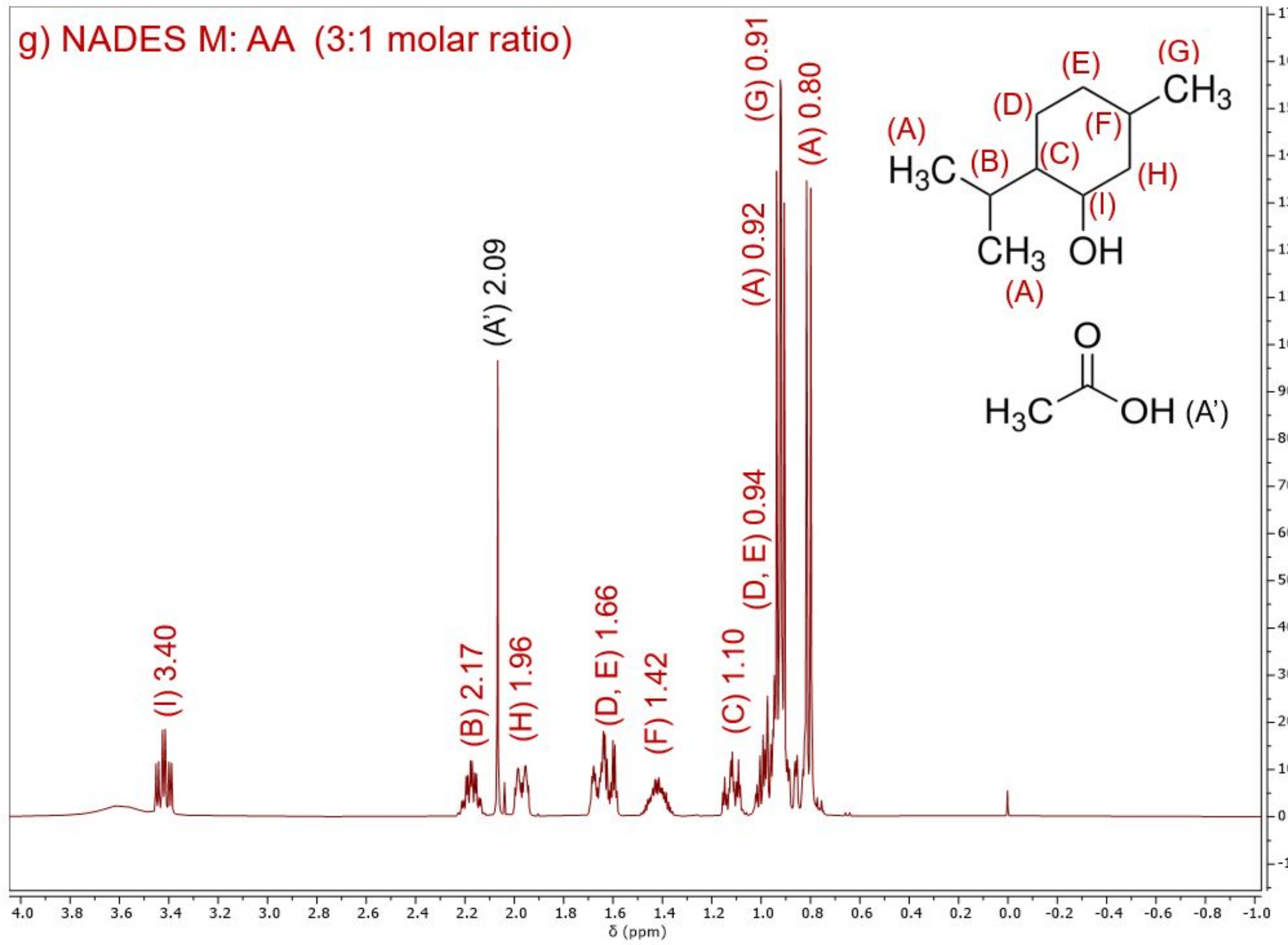
g) NADES M: AA (3:1 molar ratio)
(A') 2.09
(G) 0.91
(A) 0.80
(A) 0.92
(D, E) 0.94
(I) 3.40
(B) 2.17
(H) 1.96
(D, E) 1.66
(F) 1.42
(C) 1.10
δ (ppm)

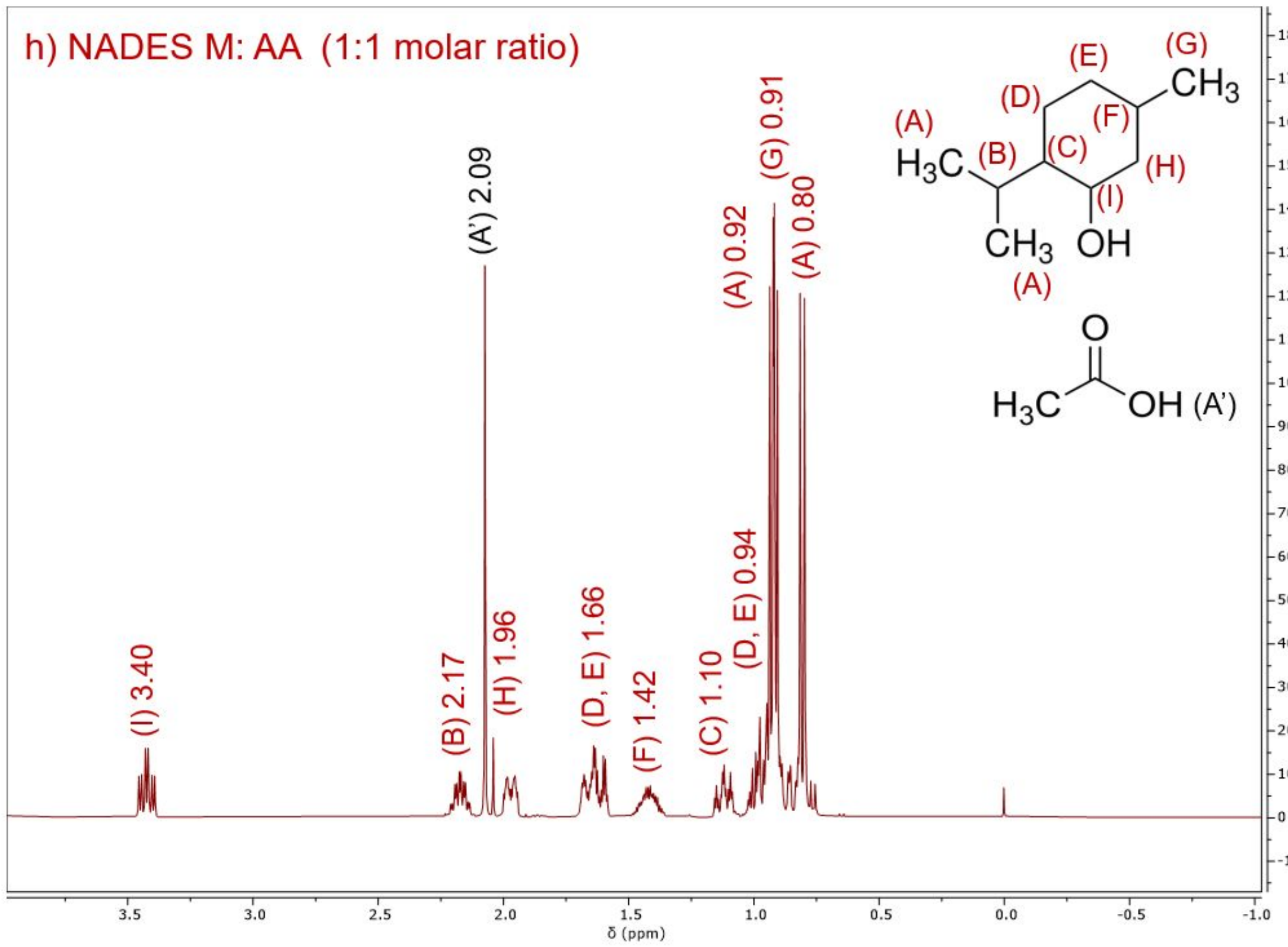
h) NADES M: AA (1:1 molar ratio)
(A') 2.09
(G) 0.91
(A) 0.92
(A) 0.80
(D, E) 0.94
(I) 3.40
(B) 2.17
(H) 1.96
(D, E) 1.66
(F) 1.42
(C) 1.10
δ (ppm)

Figure 2Sb - ^{1}H NMR spectra of (a) menthol, (b) formic acid, (c) NADES M: FA (2:1 molar ratio), and (d) rich phase after microextraction with NADES M: FA (2:1 molar ratio) under optimized conditions.

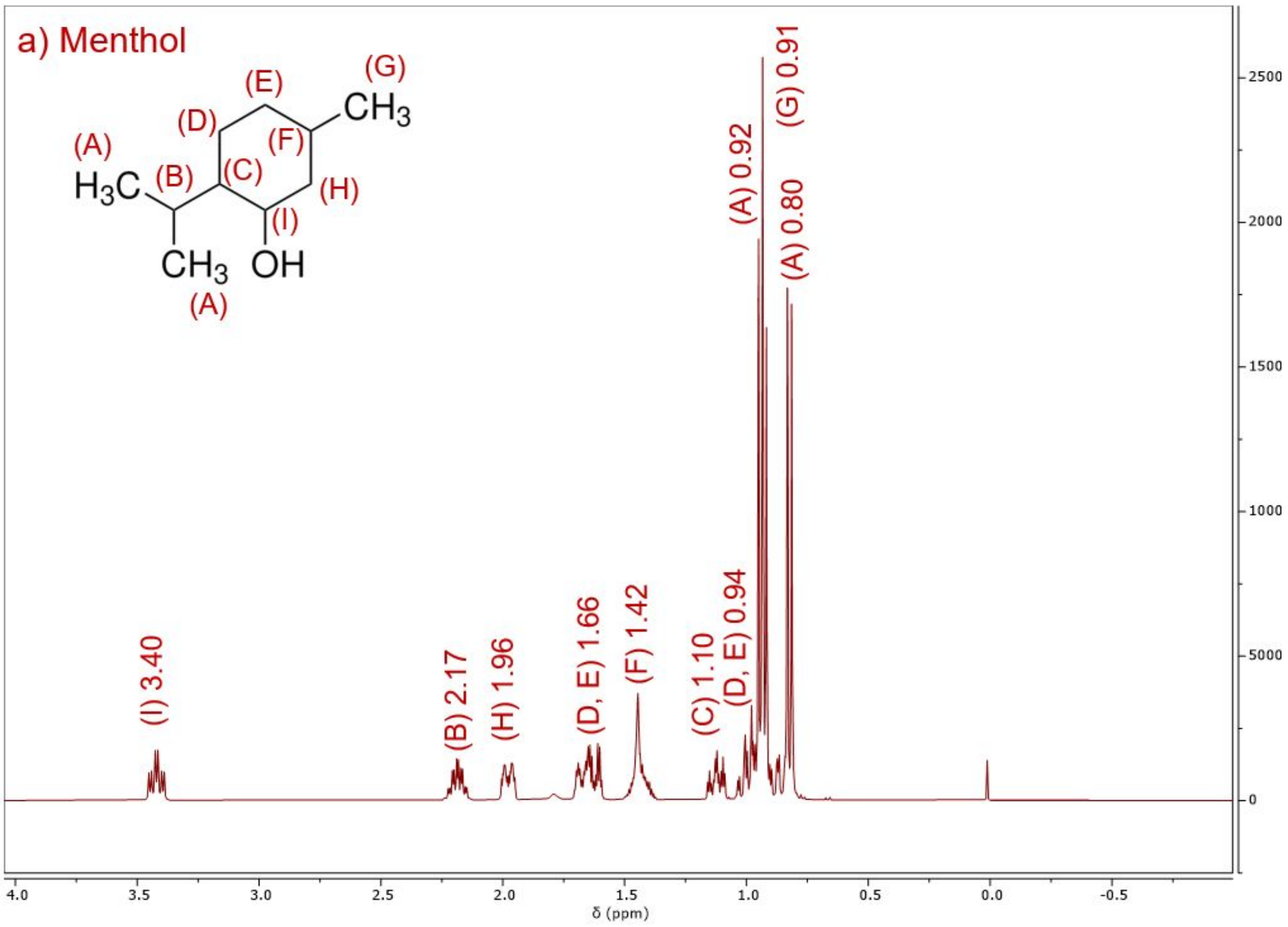

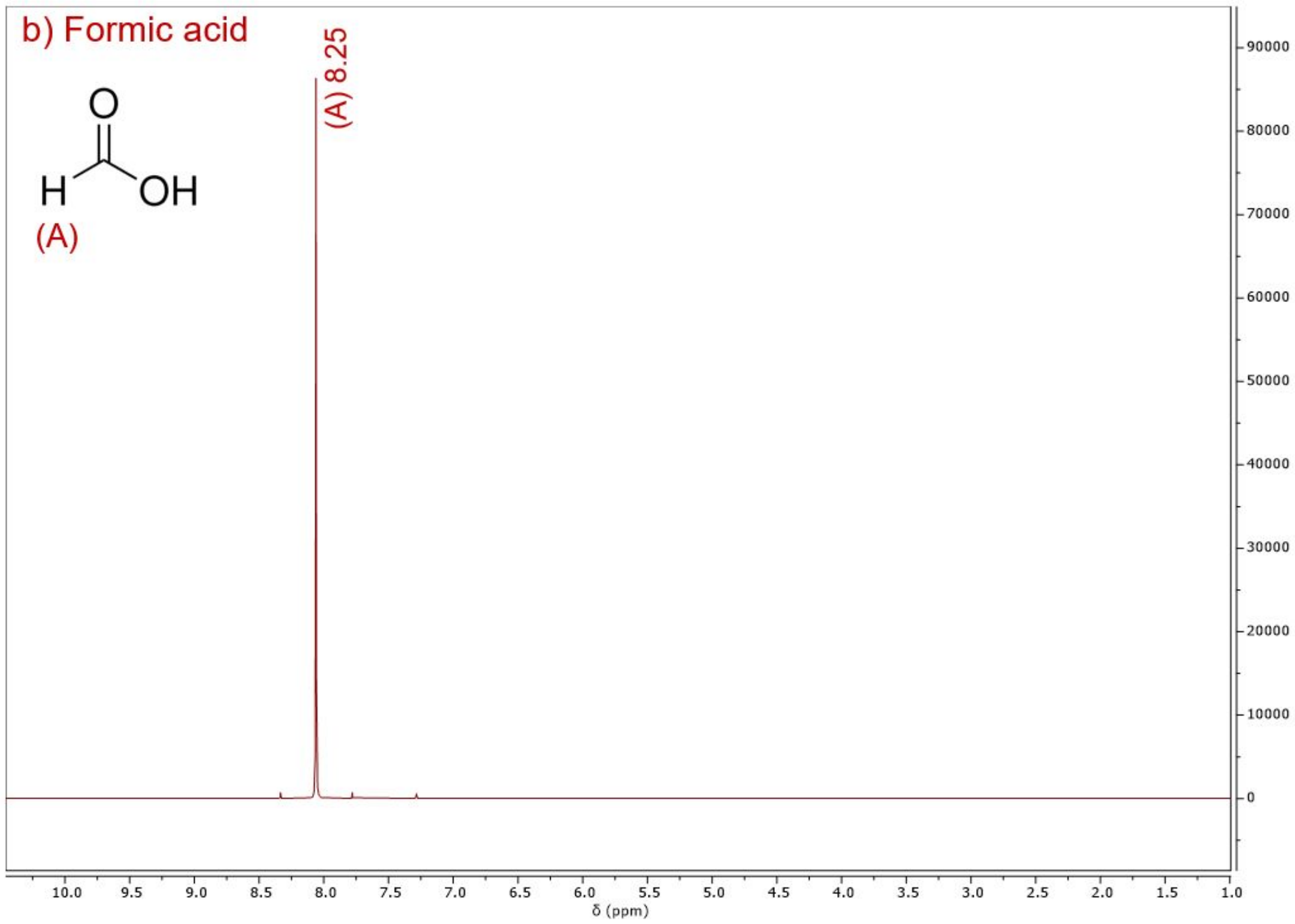

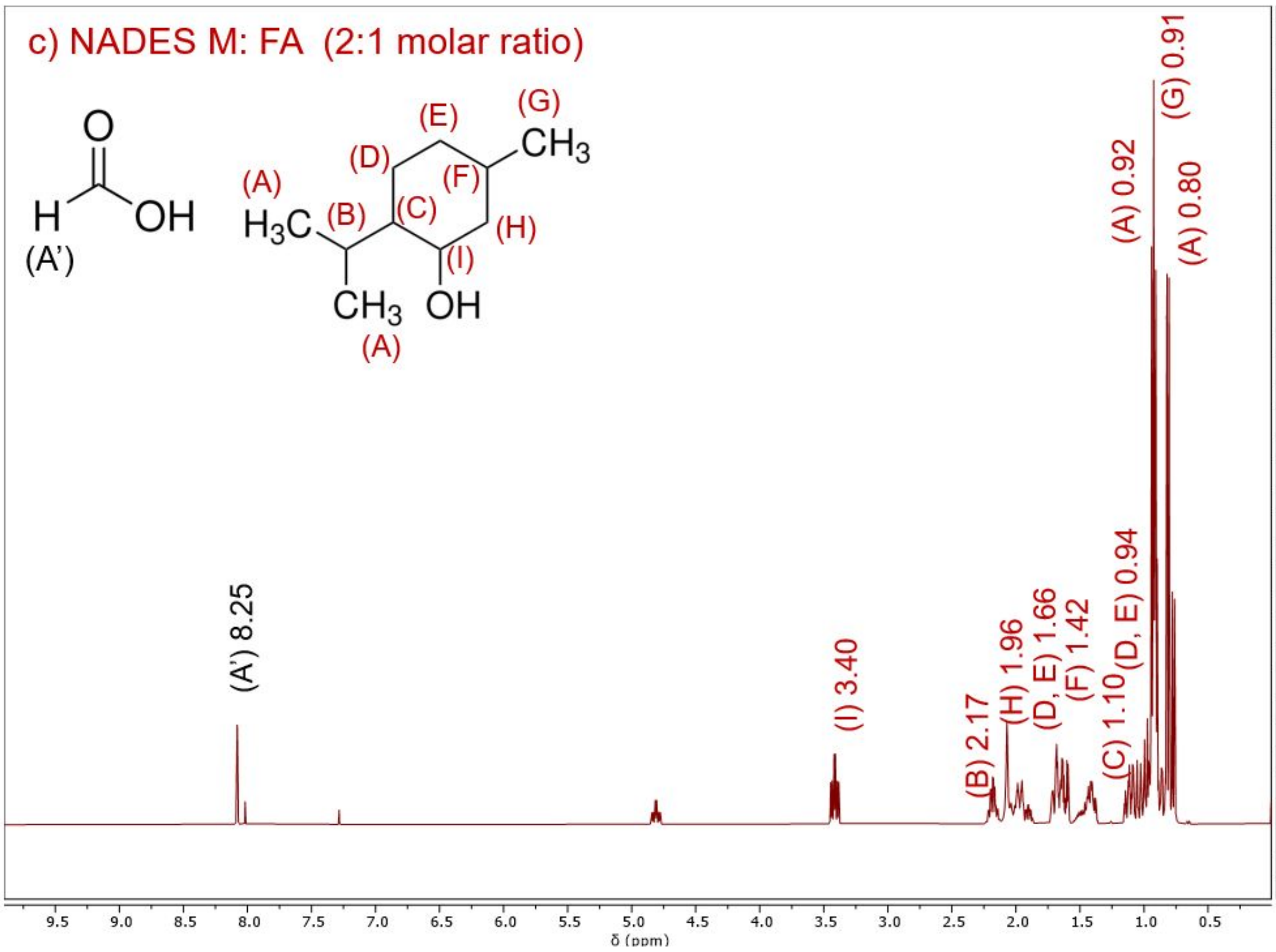
c) NADES M: FA (2:1 molar ratio)
(A') 8.25
(I) 3.40
(B) 2.17
(H) 1.96
(D, E) 1.66
(F) 1.42
(C) 1.10
(D, E) 0.94
(A) 0.92
(G) 0.91
(A) 0.80
δ (ppm)

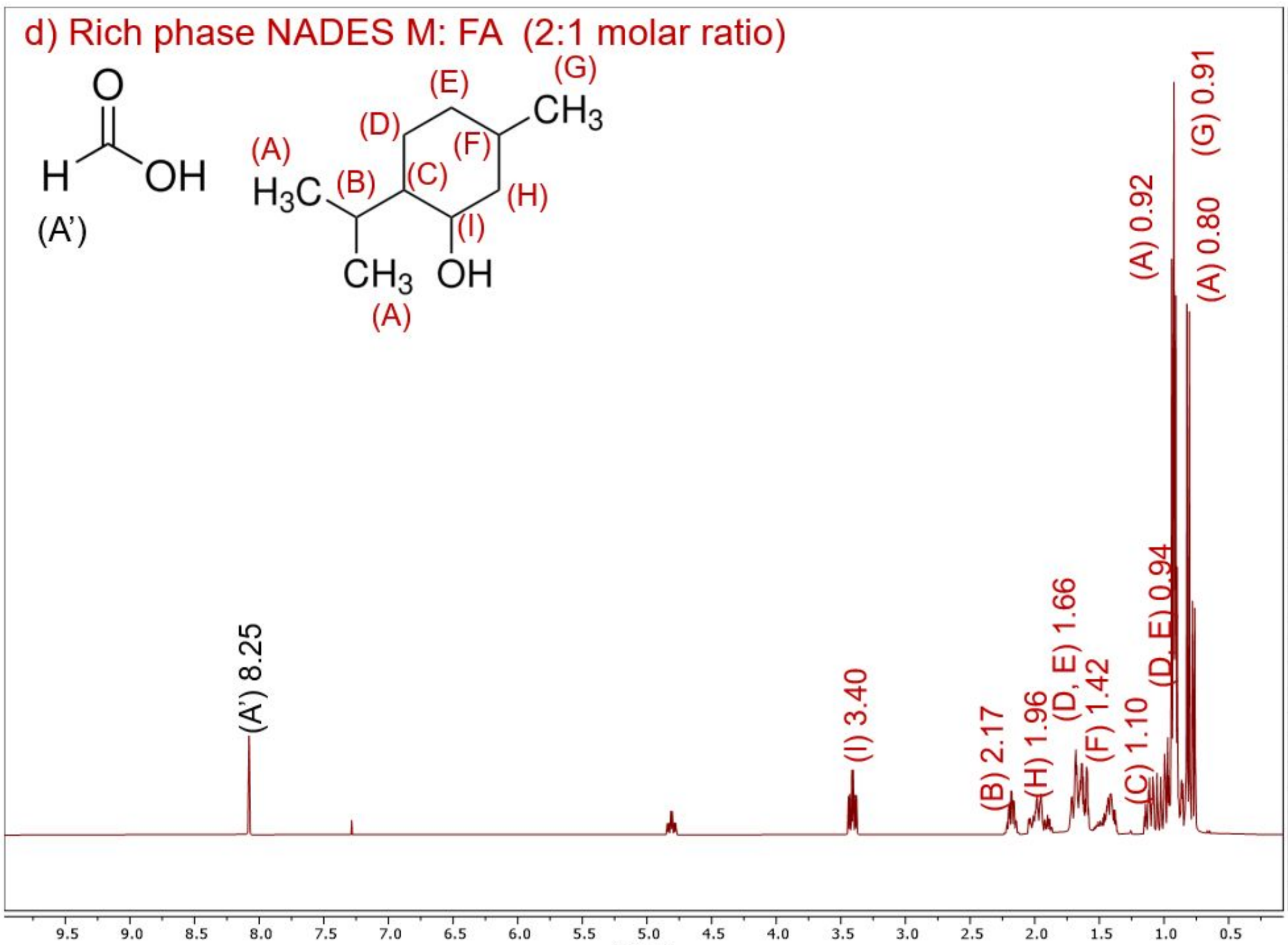
d) Rich phase NADES M: FA (2:1 molar ratio)
(A') 8.25
(I) 3.40
(B) 2.17
(H) 1.96
(D, E) 1.66
(F) 1.42
(C) 1.10
(D, E) 0.94
(A) 0.92
(G) 0.91
(A) 0.80
δ (ppm)

Figure 2Sc - ^{1}H NMR spectra of (a) menthol, (b) decanoic acid, (c) NADES M: DA (2:1 M ratio), and (d) rich phase after microextraction with NADES M: DA (2:1 molar ratio) under optimized conditions.

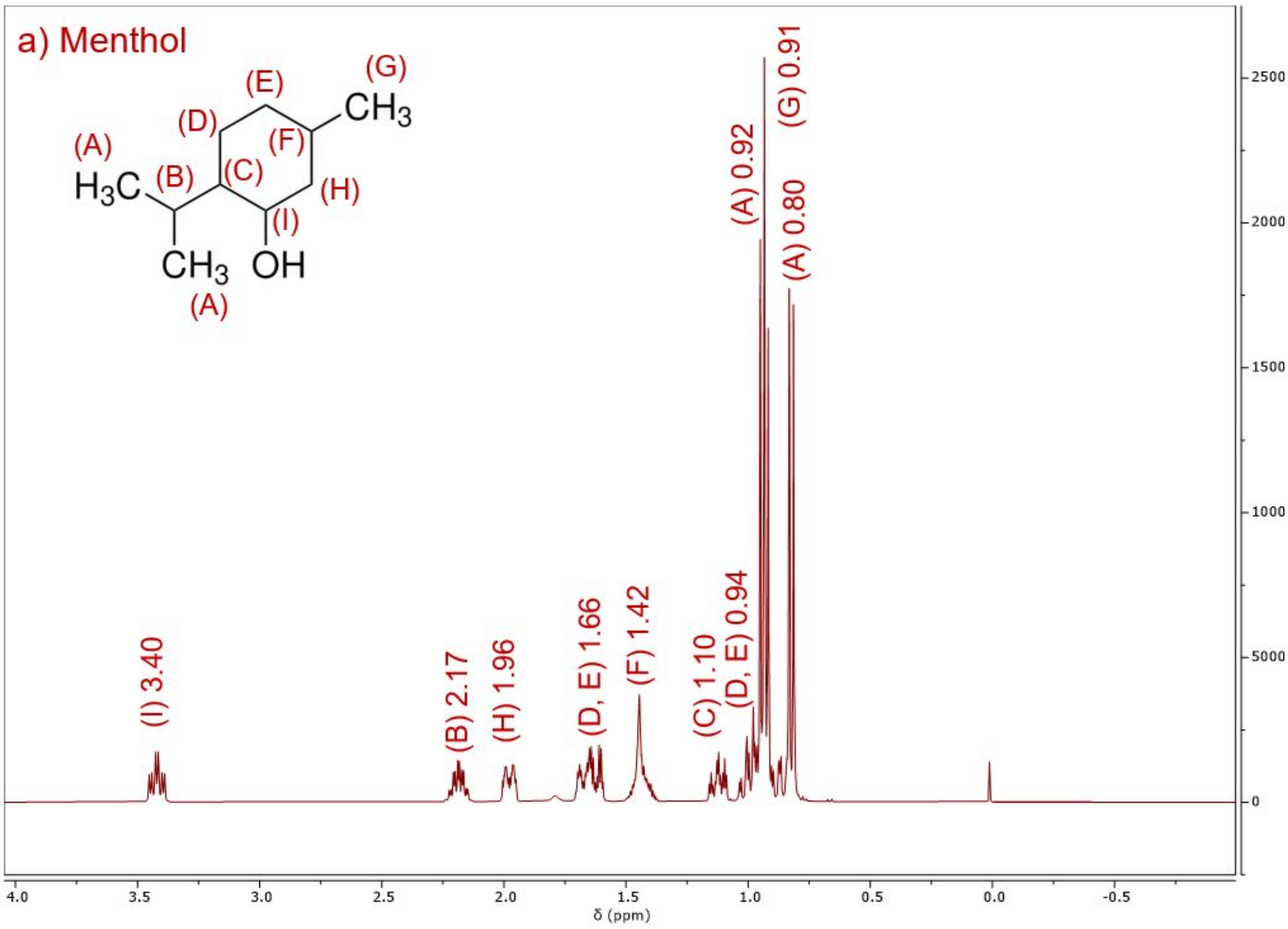

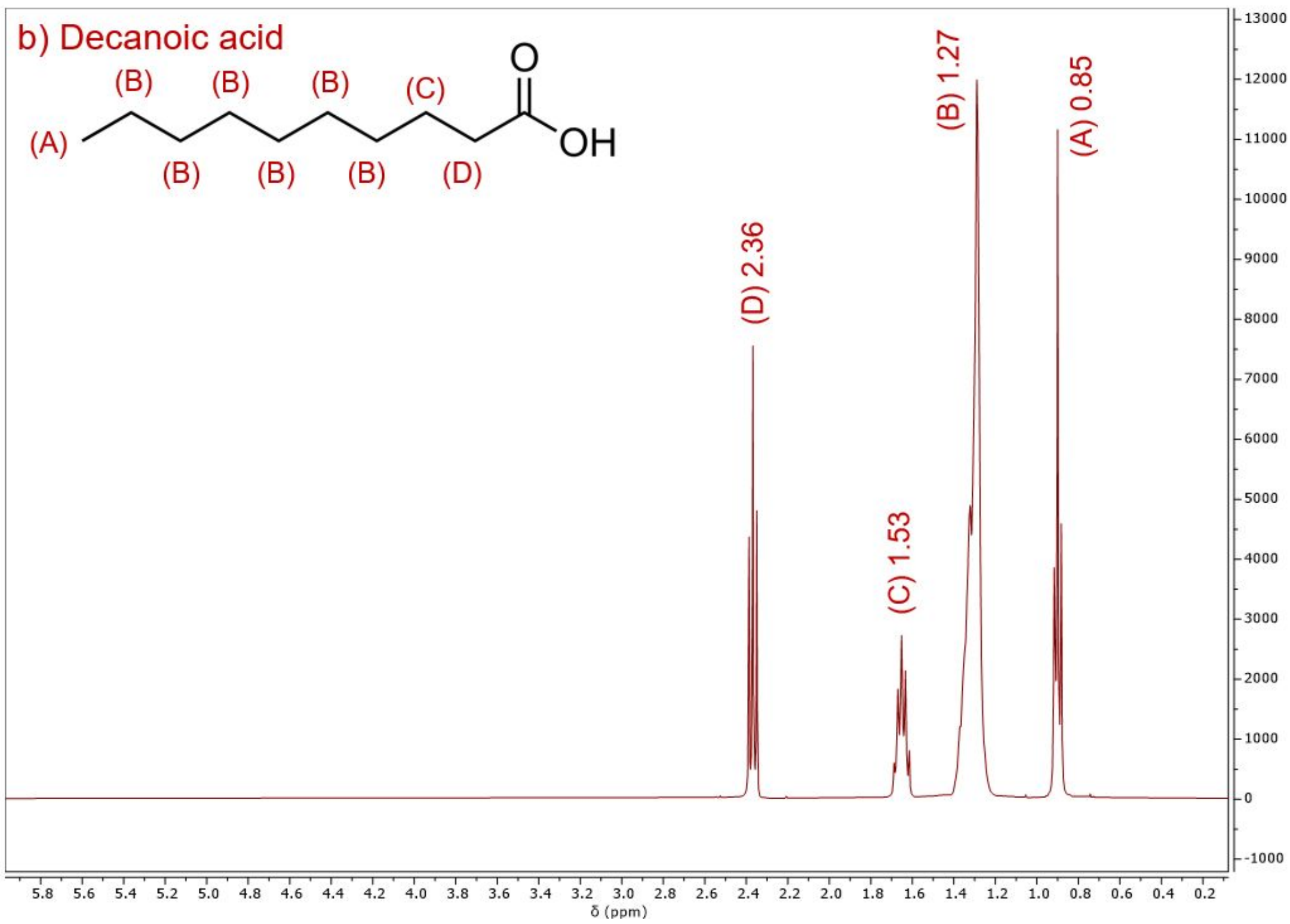

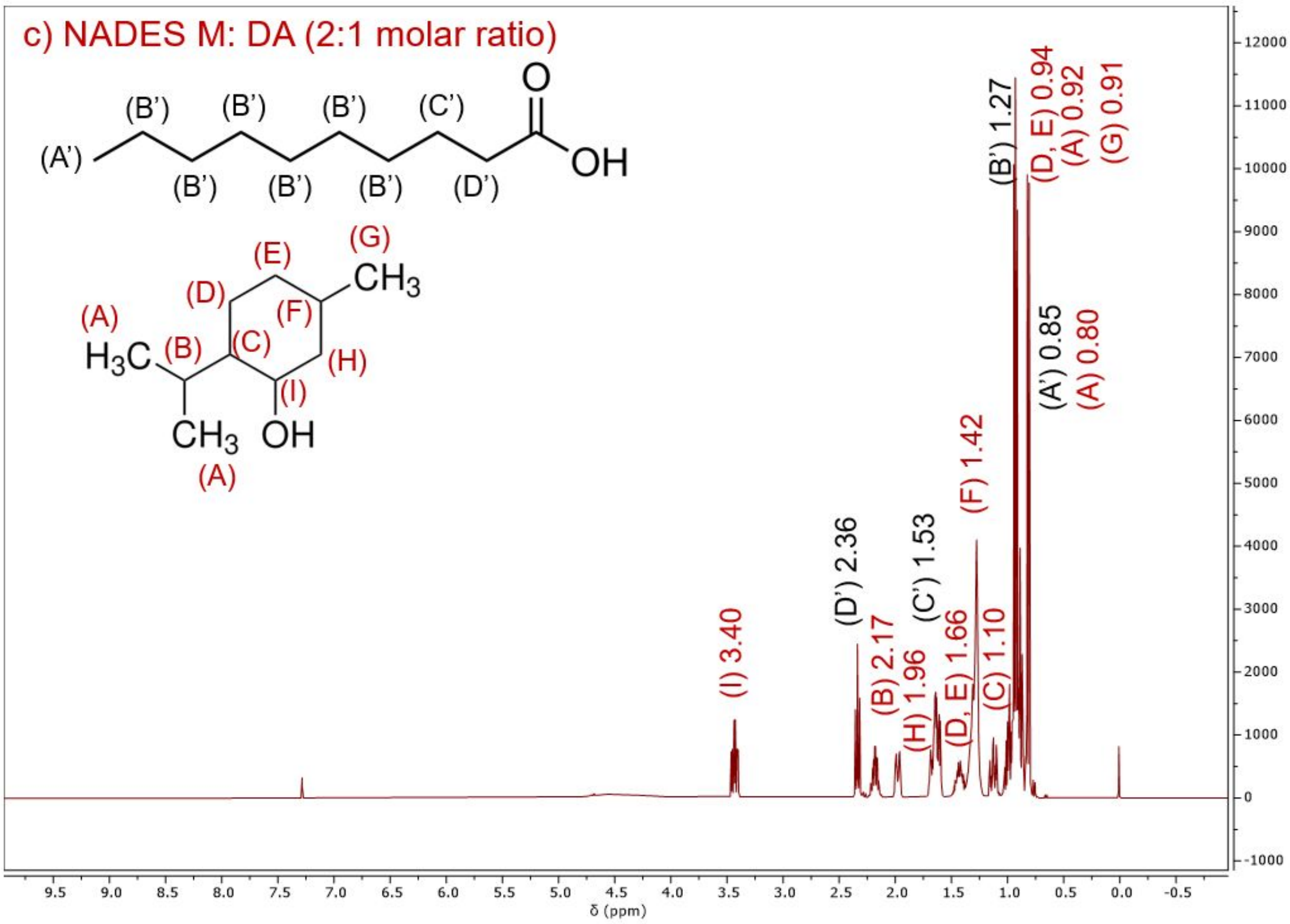
c) NADES M: DA (2:1 molar ratio)
(A') (B') (C') (D') OH O
(A) H_3C (B) (C) (D) (E) (F) (G) CH_3 (H) (I) OH CH_3 (A)
(I) 3.40
(D') 2.36
(B) 2.17
(H) 1.96
(D, E) 1.66
(C') 1.53
(F) 1.42
(B') 1.27
(C) 1.10
(D, E) 0.94
(A) 0.92
(G) 0.91
(A') 0.85
(A) 0.80
δ (ppm)

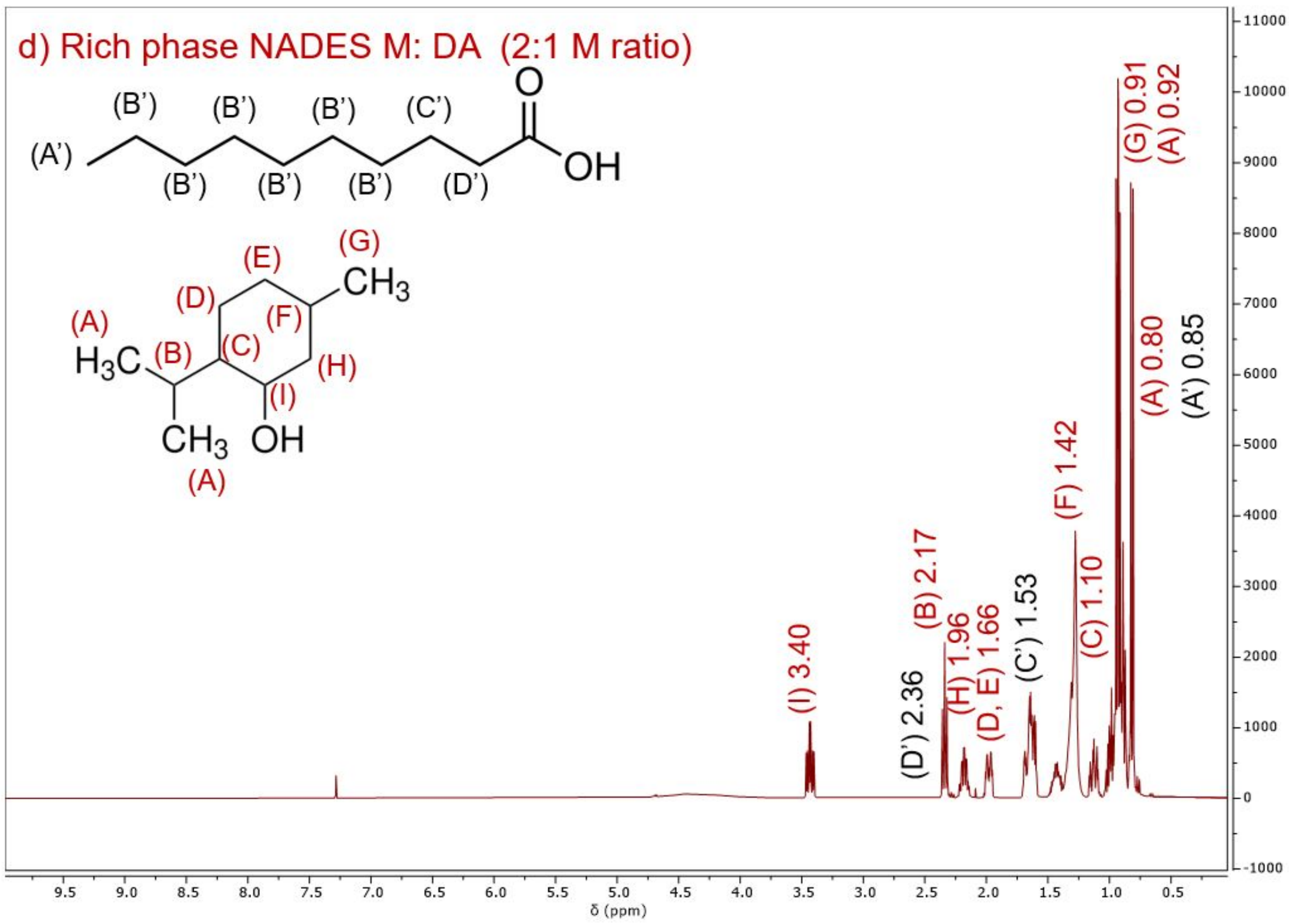
d) Rich phase NADES M: DA (2:1 M ratio)
(A') (B') (C') (D') OH O
(A) H_3C (B) (C) (D) (E) (F) (G) CH_3 (H) (I) OH CH_3 (A)
(I) 3.40
(D') 2.36
(B) 2.17
(H) 1.96
(D, E) 1.66
(C') 1.53
(F) 1.42
(C) 1.10
(G) 0.91
(A) 0.92
(A) 0.80
(A') 0.85
δ (ppm)

Figure 3S. Effect of NADES composition on microextraction of BPs (50 µg L^{-1}), condition: volume was 40 mL, 10% (w/v) of NaCl, pH 4.00 (0.01 mol L^{-1} BR Buffer), agitation time 60 s, and centrifugation at 3000 rpm for 60 s.

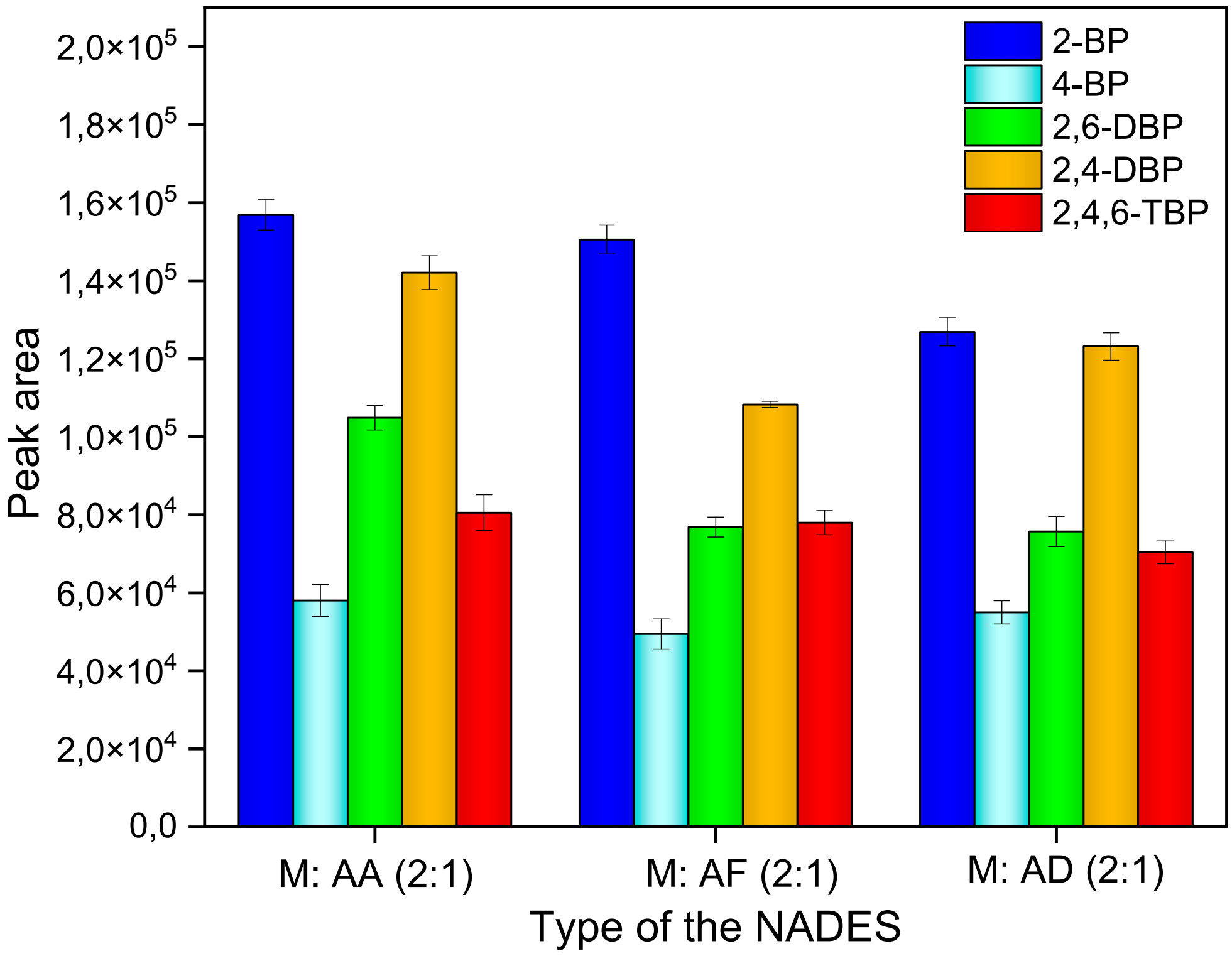

Figure 4S. Effect of molar ratio of the NADES composition on microextraction of BPs (50 µg L^{-1}), condition: volume was 40 mL, 10% (w/v) of NaCl, pH 4.00 (0.01 mol L^{-1} BR Buffer), agitation time 60 s, and centrifugation at 3000 rpm for 60 s.

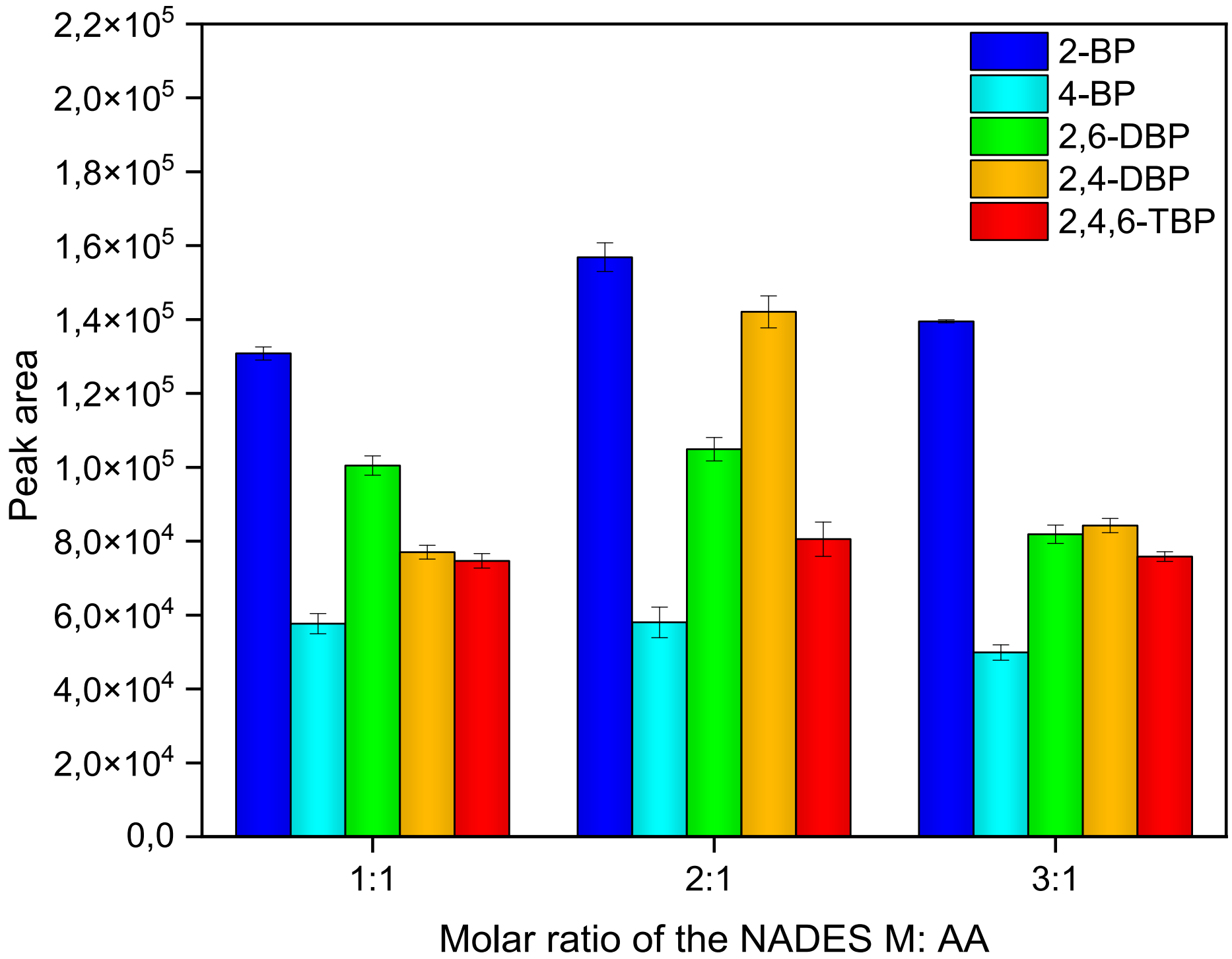

Table S2. Analysis of variance (ANOVA) from design Doehlert design

ANOVA	ANOVA	Sum of squares	Degree of freedom	Mean of squares	*F*-test
% of the salt (L)*	0.064514	1	0.064514	41.3737	0.023328
% of the salt (Q)*	0.477626	1	0.477626	306.3091	0.003249
pH (L)*	0.103180	1	0.103180	66.1711	0.014778
pH (Q)*	0.060425	1	0.060425	38.7516	0.024848
T. vortex (L)*	0.120825	1	0.120825	77.4871	0.012661
T. vortex (Q)*	0.144740	1	0.144740	92.8239	0.010602
% of the salt (L) x pH (L)	0.018196	1	0.018196	11.6694	0.076048
% of the salt (L) x T. vortex (L)	0.022733	1	0.022733	14.5792	0.062254
pH (L) x T. vortex (L)	0.117910	1	0.117910	75.6176	0.012968
Lack of fit	0.058126	3	0.019375	12.4258	0.075399
Pure error	0.003119	2	0.001559		
Total	1.008690	14			

L: linear; Q: Quadratic

Figure 6S. Analytical curve with and without the preconcentration step

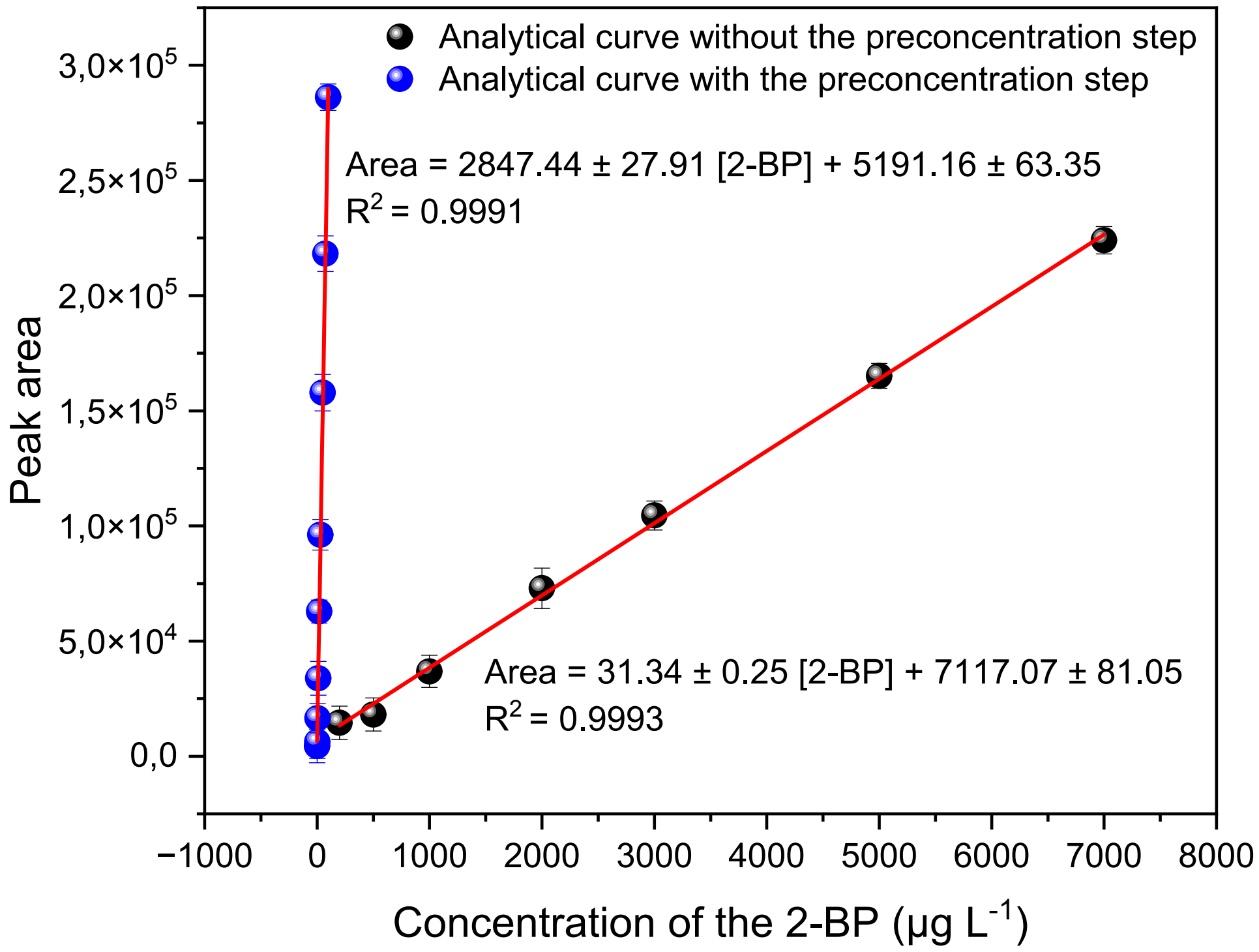

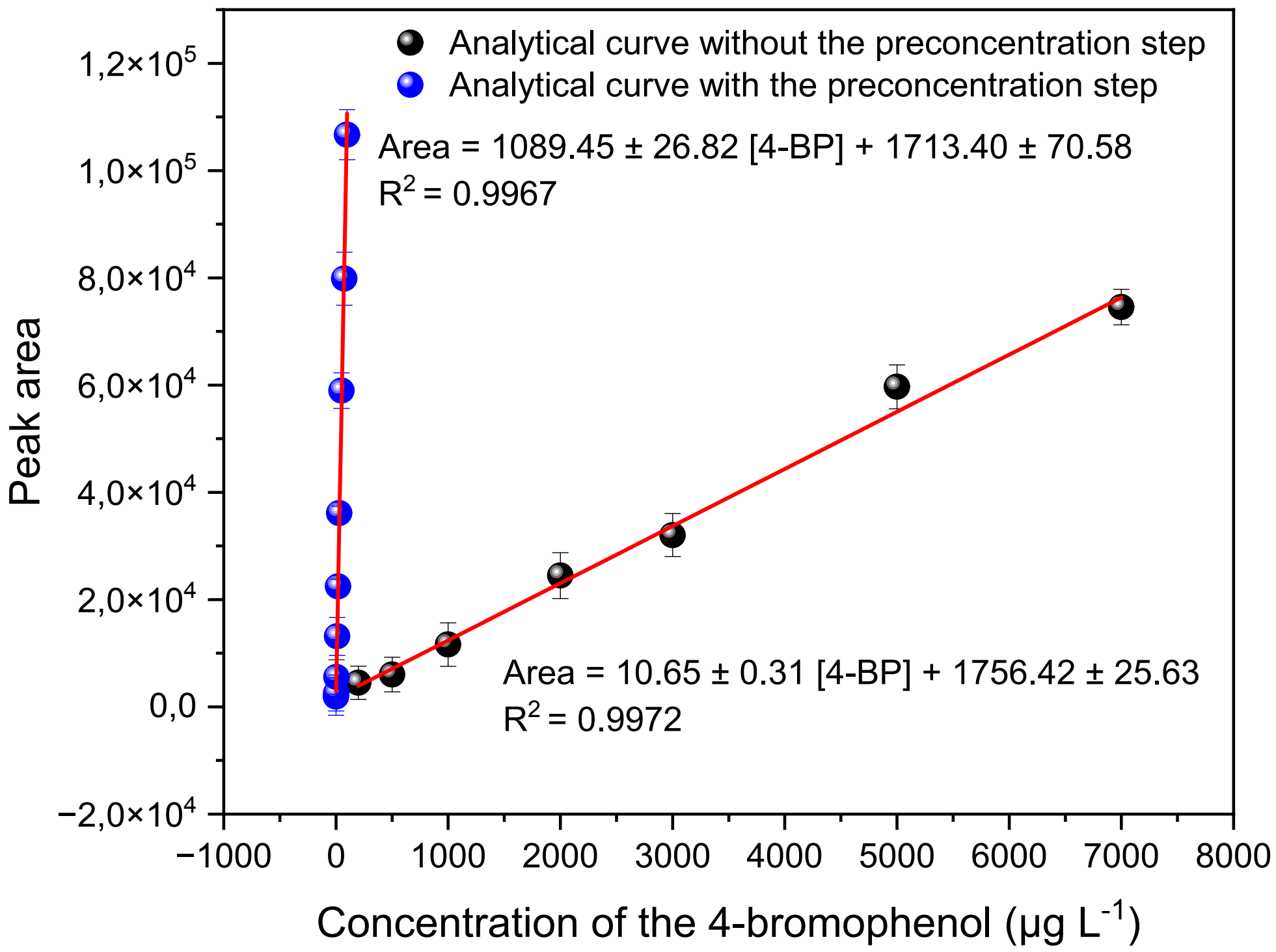

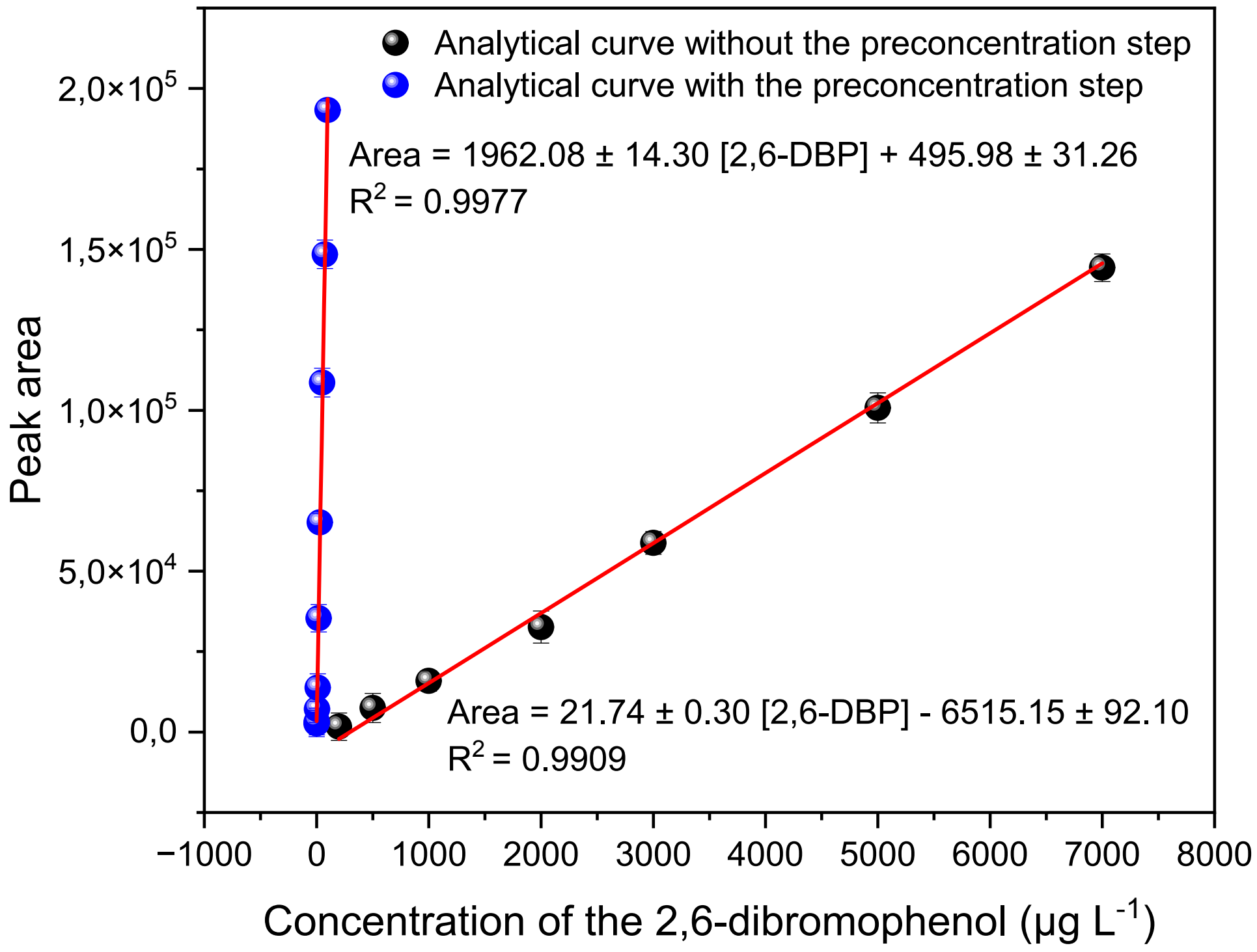

Analytical curve without the preconcentration step
Analytical curve with the preconcentration step
Area = 1962.08 ± 14.30 [2,6-DBP] + 495.98 ± 31.26
R2 = 0.9977
Area = 21.74 ± 0.30 [2,6-DBP] - 6515.15 ± 92.10
R2 = 0.9909
Peak area
2,0×10^5
1,5×10^5
1,0×10^5
5,0×10^4
0,0
−1000
0
1000
2000
3000
4000
5000
6000
7000
8000
Concentration of the 2,6-dibromophenol (μg L-1)

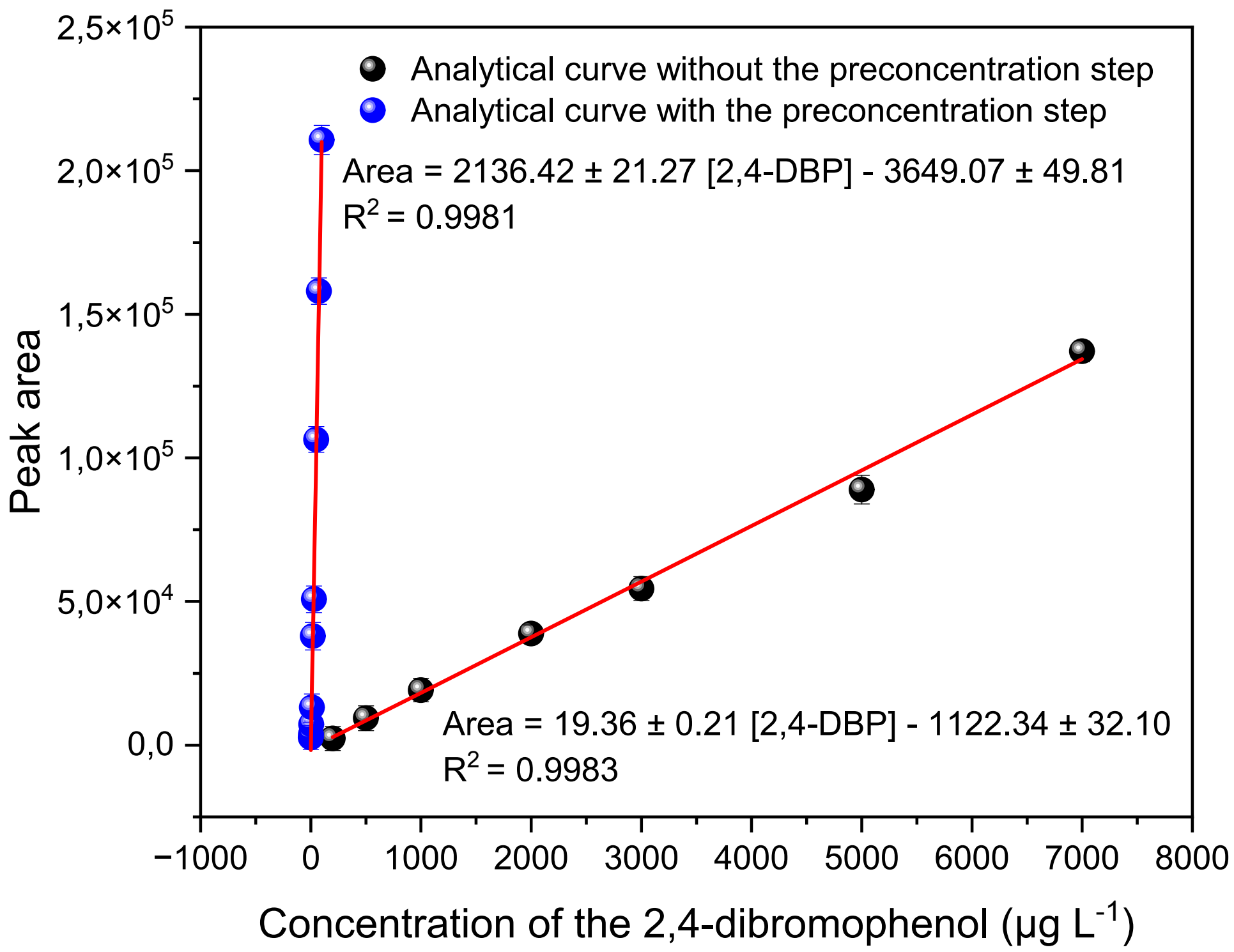

Analytical curve without the preconcentration step
Analytical curve with the preconcentration step
Area = 2136.42 ± 21.27 [2,4-DBP] - 3649.07 ± 49.81
R2 = 0.9981
Area = 19.36 ± 0.21 [2,4-DBP] - 1122.34 ± 32.10
R2 = 0.9983
Peak area
2,5×10^5
2,0×10^5
1,5×10^5
1,0×10^5
5,0×10^4
0,0
−1000
0
1000
2000
3000
4000
5000
6000
7000
8000
Concentration of the 2,4-dibromophenol (μg L-1)

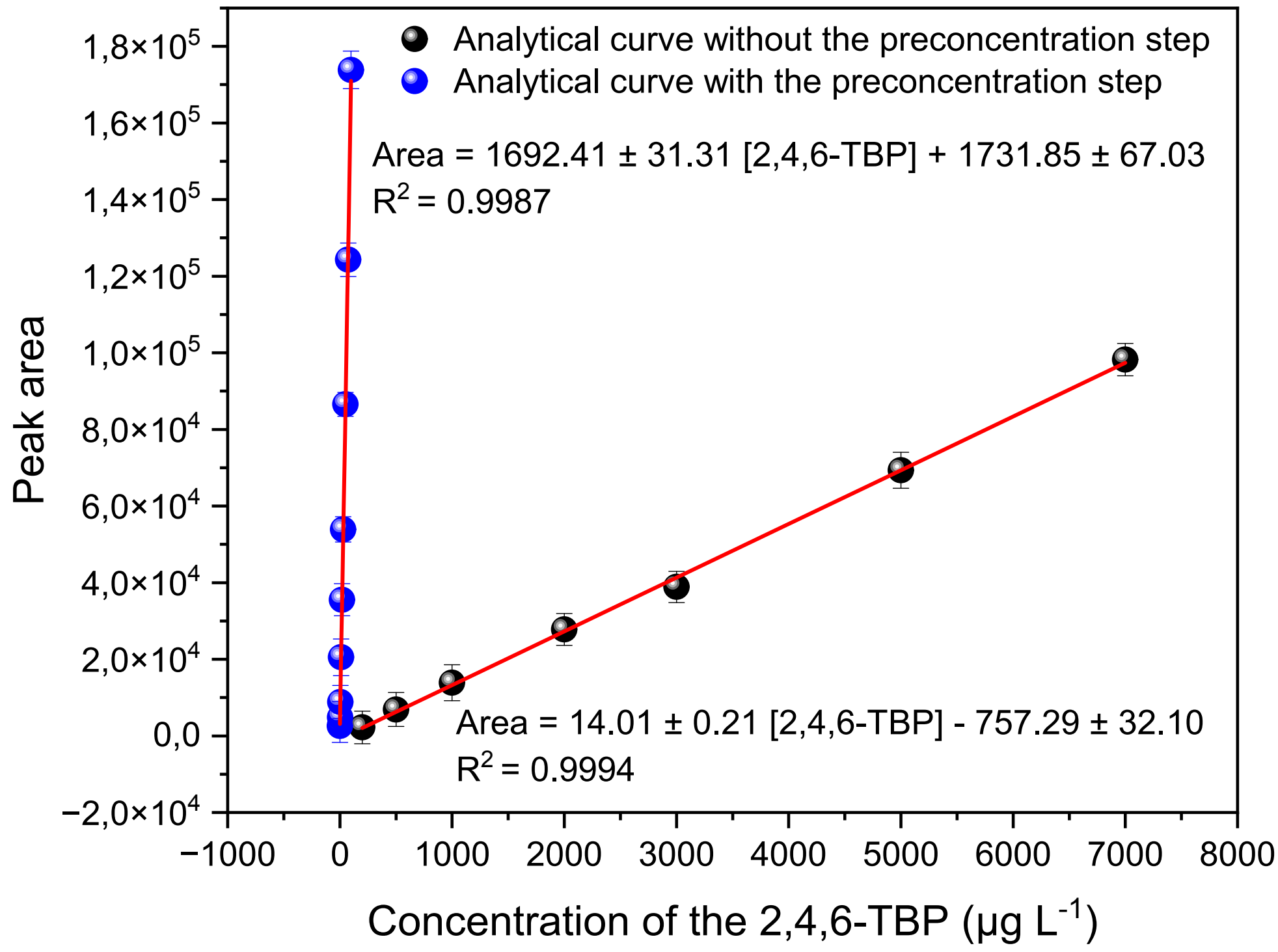

Table S3. Intra-day precision and intermediate precision

Analyte	Intra-day precision (n= 5)			Intermediate precision (n= 5)		
	5 μg L^{-1}	20 μg L^{-1}	75 μg L^{-1}	5 μg L^{-1}	20 μg L^{-1}	75 μg L^{-1}
2-BP	3.95	3.65	2.33	3.60	3.26	1.69
4-BP	1.16	4.03	3.34	2.37	5.00	3.42
2,6-DBP	2.03	2.98	2.81	3.71	5.11	2.11
2,4-DBP	3.95	4.40	1.50	4.07	4.70	2.10
2,4,6-TBP	4.60	1.67	1.14	3.31	2.56	3.27

Figure 7S. Product ion spectra obtained from 2-BP, 4-BP, and 2,6-DPB.

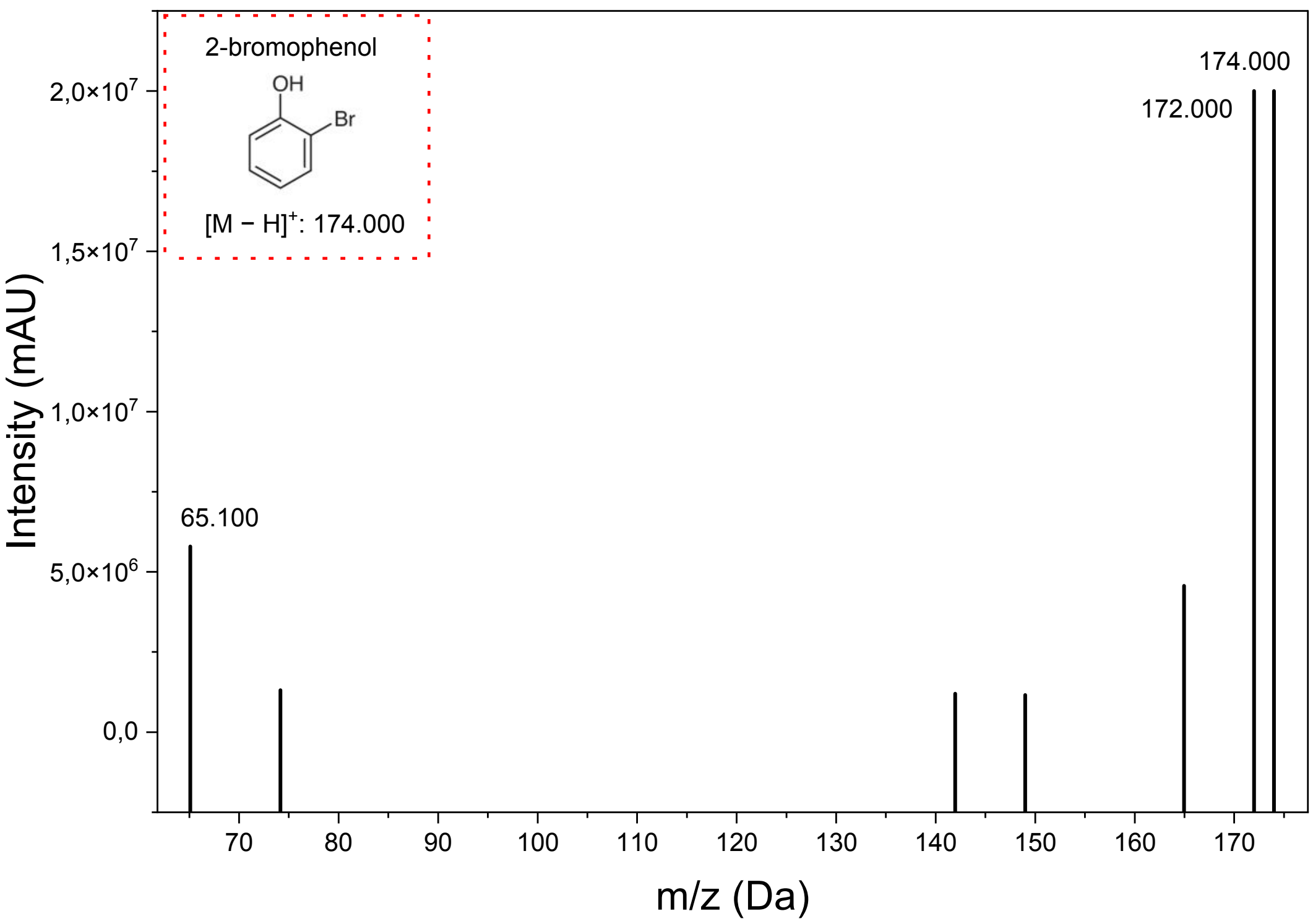

2-bromophenol
OH
Br
[M − H]+: 174.000
174.000
172.000
65.100
Intensity (mAU)
2,0×10^7
1,5×10^7
1,0×10^7
5,0×10^6
0,0
70
80
90
100
110
120
130
140
150
160
170
m/z (Da)

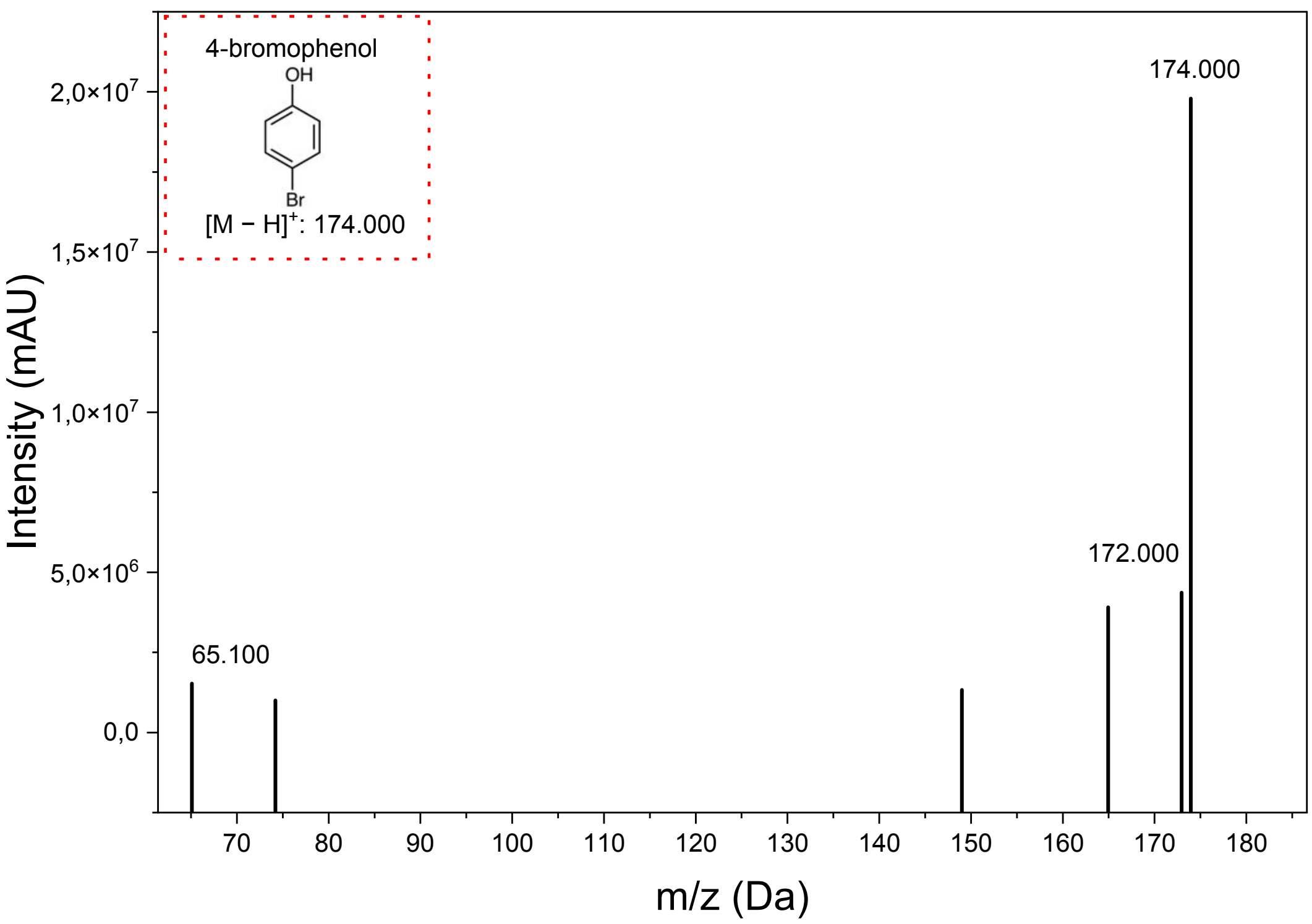

4-bromophenol
OH
Br
[M − H]+: 174.000
174.000
172.000
65.100
Intensity (mAU)
2,0×10^7
1,5×10^7
1,0×10^7
5,0×10^6
0,0
70
80
90
100
110
120
130
140
150
160
170
180
m/z (Da)

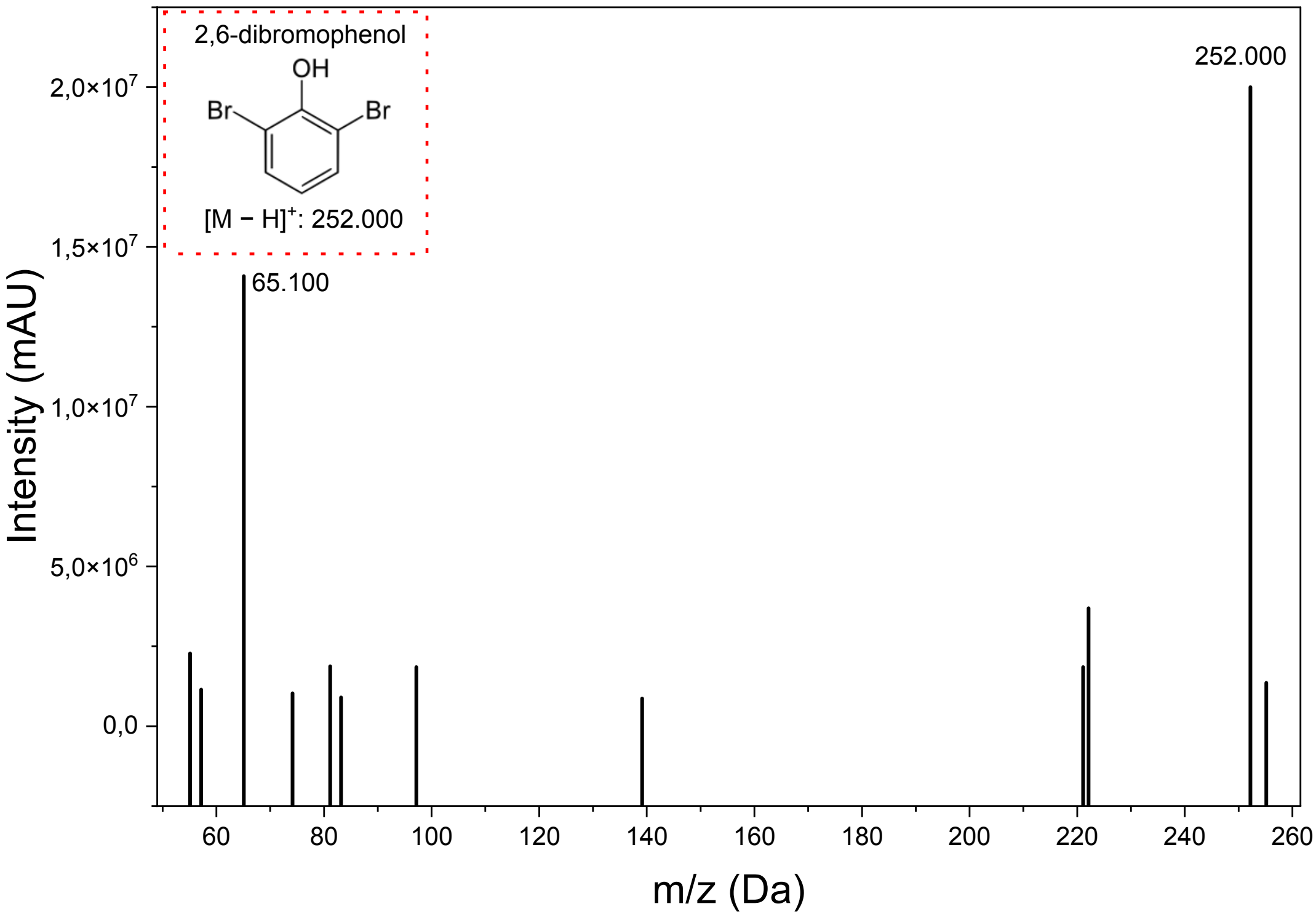

2,6-dibromophenol
OH
Br
Br
[M − H]+: 252.000
252.000
65.100
2,0×10^7
1,5×10^7
1,0×10^7
5,0×10^6
0,0
Intensity (mAU)
60
80
100
120
140
160
180
200
220
240
260
m/z (Da)

Table S4. BAGI and AGREE evaluation for the BPs determination methods using liquid-phase-based extraction methods

BAGI		AGREE		Reference
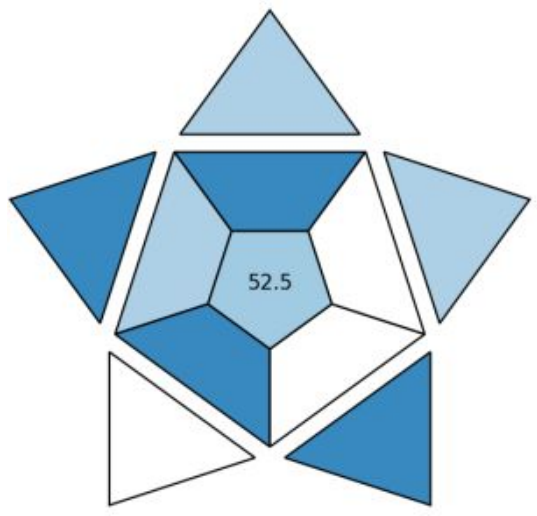	Multiple elements within the same class analysis. The extraction method is time-consuming and analyzes only a few samples per hour.	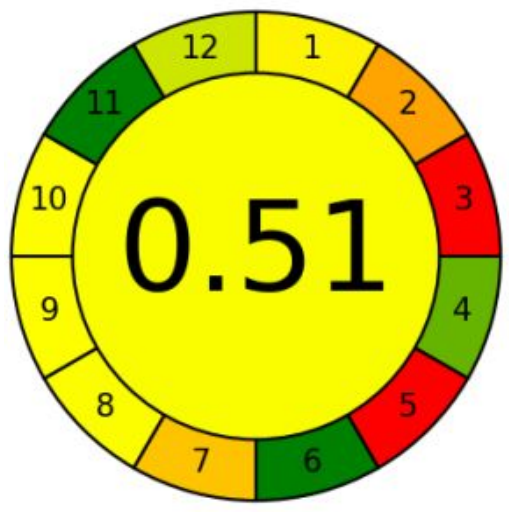	No derivatization and analysis of various BPs. Offline analysis and sample preparation are not miniaturized.	[3]
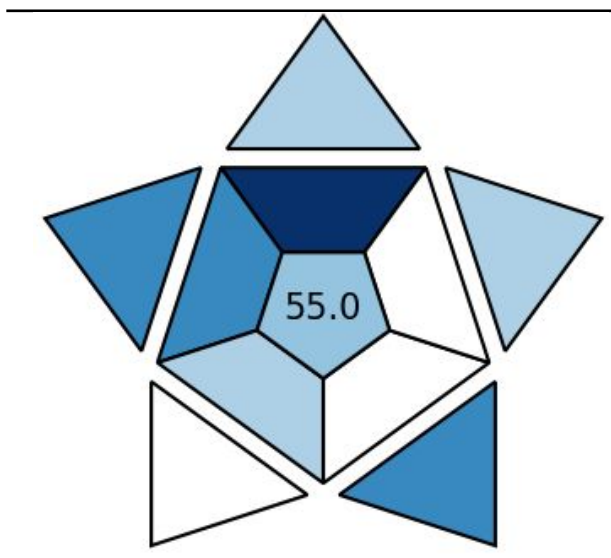	GC-MS for quantitative and confirmatory analysis. The extraction method is time-consuming, and the sample preparation requires multiple steps.	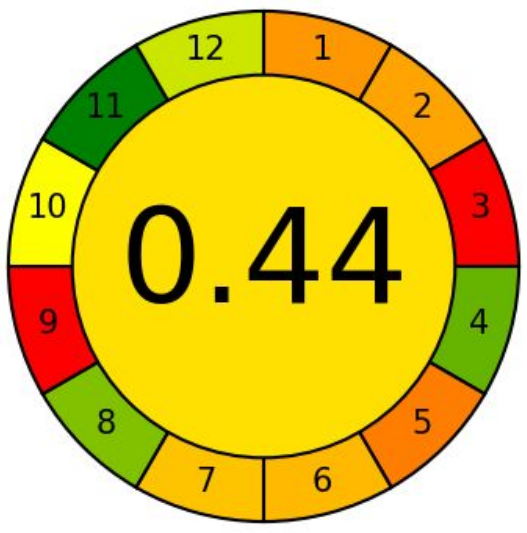	Multiple analyte analyses in samples are complex. Requires derivatization (CAS Number: 25561-30-2).	[10]
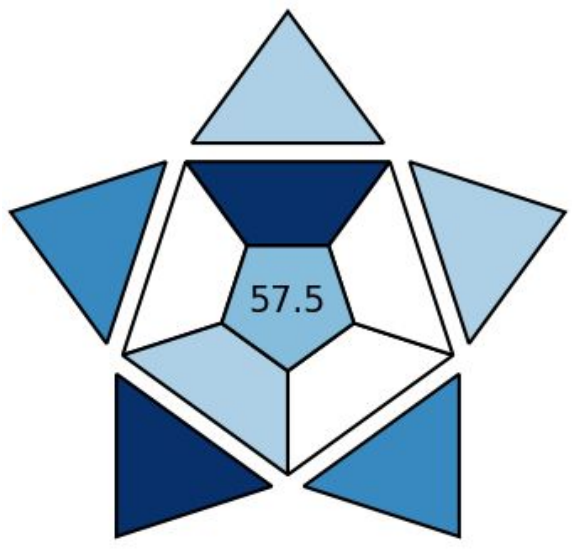	Quantitative and confirmatory analysis using GC-MS. However, analysis of only one analyte.	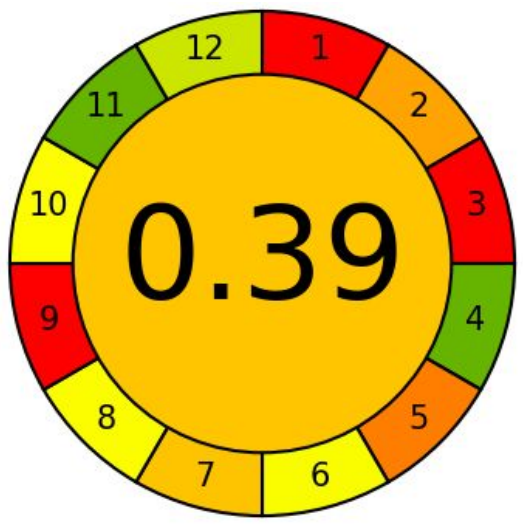	Instrumental sensitivity, however, requires derivatization (CAS Number: 108-24-7) and high energy consumption by instrumental technique.	[13]
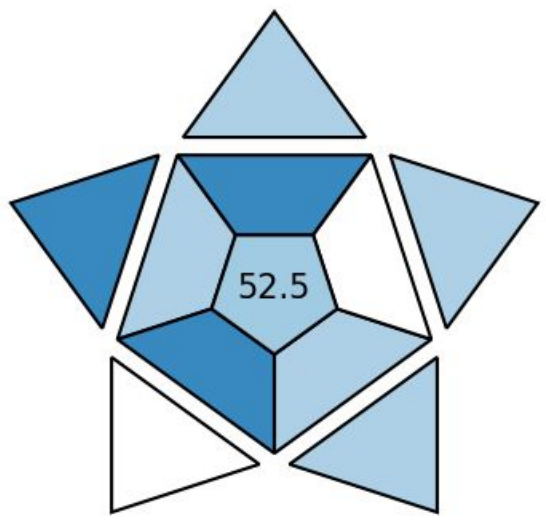	Determines several analytes per analysis, despite the low analytical sensitivity.	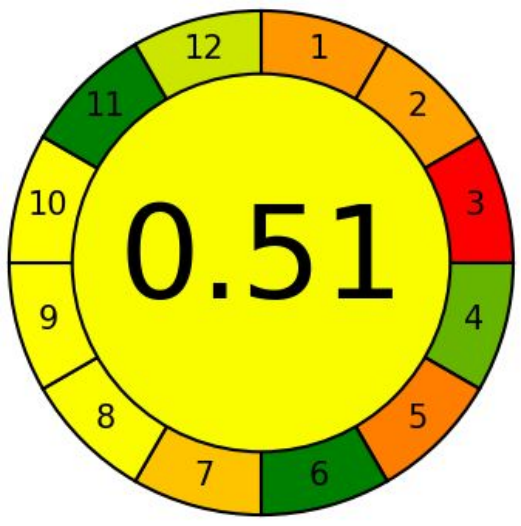	It does not require derivatization; however, the extraction method is elaborate and time-consuming.	[56]
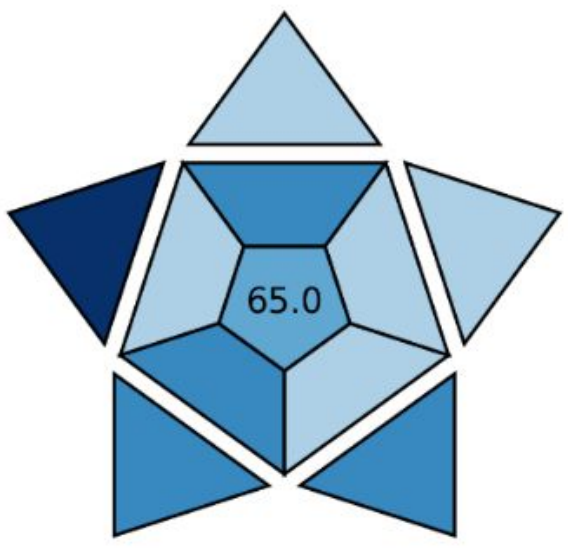	Determination of various analytes by analysis and preconcentration methods prepared with common laboratory reagents, overcoming the low sensitivity of the instrumental technique.	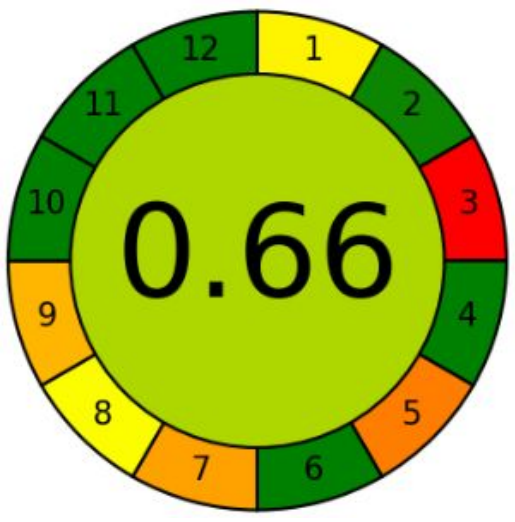	Use of environmentally friendly solvents, determination of 5 BPs, and a simple and fast sample preparation method. The instrumental technique requires a large consumption of solvents and energy.	This study
